# What is open peer review? A systematic review

**DOI:** 10.12688/f1000research.11369.2

**Published:** 2017-08-31

**Authors:** Tony Ross-Hellauer

**Affiliations:** 1 Göttingen State and University Library, University of Göttingen, Göttingen, 37073, Germany

**Keywords:** open peer review, Open Science, scholarly communication, research evaluation, publishing

## Abstract

**Background**: “Open peer review” (OPR), despite being a major pillar of Open Science, has neither a standardized definition nor an agreed schema of its features and implementations. The literature reflects this, with numerous overlapping and contradictory definitions. While for some the term refers to peer review where the identities of both author and reviewer are disclosed to each other, for others it signifies systems where reviewer reports are published alongside articles. For others it signifies both of these conditions, and for yet others it describes systems where not only “invited experts” are able to comment. For still others, it includes a variety of combinations of these and other novel methods.

**Methods**: Recognising the absence of a consensus view on what open peer review is, this article undertakes a systematic review of definitions of “open peer review” or “open review”, to create a corpus of 122 definitions. These definitions are systematically analysed to build a coherent typology of the various innovations in peer review signified by the term, and hence provide the precise technical definition currently lacking.

**Results**: This quantifiable data yields rich information on the range and extent of differing definitions over time and by broad subject area. Quantifying definitions in this way allows us to accurately portray exactly how ambiguously the phrase “open peer review” has been used thus far, for the literature offers 22 distinct configurations of seven traits, effectively meaning that there are 22 different definitions of OPR in the literature reviewed.

**Conclusions**: I propose a pragmatic definition of open peer review as an umbrella term for a number of overlapping ways that peer review models can be adapted in line with the aims of Open Science, including making reviewer and author identities open, publishing review reports and enabling greater participation in the peer review process.

## Introduction

  “Open review and open peer review are new terms for evolving phenomena. They don’t have precise or technical definitions. No matter how they’re defined, there’s a large area of overlap between them. If there’s ever a difference, some kinds of open review accept evaluative comments from any readers, even anonymous readers, while other kinds try to limit evaluative comments to those from ”peers“ with expertise or credentials in the relevant field. But neither kind of review has a special name, and I think each could fairly be called “open review” or “open peer review”.” - Peter Suber, email correspondence, 2007 ^1^.

As with other areas of “open science” ( Pontika *et al.*, 2015), “open peer review” (OPR) is a hot topic, with a rapidly growing literature that discusses it. Yet, as has been consistently noted ( Ford, 2013; Hames, 2014; Ware, 2011), OPR has neither a standardized definition, nor an agreed schema of its features and implementations. The literature reflects this, with a myriad of overlapping and often contradictory definitions. While the term is used by some to refer to peer review where the identities of both author and reviewer are disclosed to each other, for others it signifies systems where reviewer reports are published alongside articles. For others it signifies both of these conditions, and for yet others it describes systems where not only “invited experts” are able to comment. For still others, it includes a variety of combinations of these and other novel methods. The previous major attempt to resolve these elements systematically to provide a unified definition ( Ford, 2013), discussed later, unfortunately ultimately confounds rather than resolves these issues.

In short, things have not improved much since Suber made his astute observation. This continuing imprecision grows more problematic over time, however. As Mark Ware notes, “it is not always clear in debates over the merits of OPR exactly what is being referred to” ( Ware, 2011). Differing flavours of OPR include independent factors (open identities, open reports, open participation, etc.), which have no necessary connection to each other, and very different benefits and drawbacks. Evaluation of the efficacy of these differing variables and hence comparison between differing systems is therefore problematic. Discussions are potentially side-tracked when claims are made for the efficacy of “OPR” in general, despite critique usually being focussed on one element or distinct configuration of OPR. It could even be argued that this inability to define terms is to blame for the fact that, as Nicholas Kriegskorte has pointed out, “we have yet to develop a coherent shared vision for “open evaluation” (OE), and an OE movement comparable to the OA movement” ( Kriegeskorte, 2012).

To resolve this, I undertake a systematic review of the definitions of “open peer review” or “open review”, to create a corpus of more than 120 definitions. These definitions have been systematically analysed to build a coherent typology of the many different innovations in peer review signified by the term, and hence provide the precise technical definition that is currently lacking. This quantifiable data yields rich information on the range and extent of differing definitions over time and by broad subject area. Based on this work, I propose a pragmatic definition of OPR as an umbrella term for a number of overlapping ways that peer review models can be adapted in line with the aims of Open Science, including making reviewer and author identities open, publishing review reports and enabling greater participation in the peer review process.

## Background

### 1. Problems with peer review

Peer review is the formal quality assurance mechanism whereby scholarly manuscripts (e.g. journal articles, books, grant applications and conference papers) are made subject to the scrutiny of others, whose feedback and judgements are then used to improve works and make final decisions regarding selection (for publication, grant allocation or speaking time). Peer review usually performs two distinct functions: (1) technical evaluation of the validity or soundness of a work in its methodology, analysis and argumentation (answering the question “is it good scholarship?”), and (2) assisting editorial selection by assessing the novelty or expected impact of a work (“is it exciting, innovative or important scholarship?”, “is it right for this journal, conference or funding call?”). The two processes need not be entwined, and some journals such as PLOS ONE and PeerJ, have begun to adopt models where reviewers are asked to focus only on technical soundness.

This broad system is perhaps more recent than one might expect, with its main formal elements only in general use since the mid-twentieth century in scientific publishing ( Spier, 2002). Researchers agree that peer review *per se* is necessary, but most find the current model sub-optimal. Ware’s 2008 survey, for example, found that an overwhelming majority (85%) agreed that “peer review greatly helps scientific communication” and that even more (around 90%) said their own last published paper had been improved by peer review. Yet almost two thirds (64%) declared that they were satisfied with the current system of peer review, and less than a third (32%) believed that this system was the best possible ( Ware, 2008). A recent follow-up study by the same author reported a slight increase in the desire for improvements in peer review ( Ware, 2016)

Widespread beliefs that the current model is sub-optimal can be attributed to the various ways in which traditional peer review has been subject to criticism. These criticisms apply to differing levels, with some concerning the work of peer reviewers themselves, and others more concerned with editorial decisions based upon or affecting peer review. I next give a brief overview of these various criticisms of traditional peer review:

- **Unreliability and inconsistency:** Reliant upon the vagaries of human judgement, the objectivity, reliability, and consistency of peer review are subject to question. Studies show reviewers’ views tend to show very weak levels of agreement ( Kravitz *et al.*, 2010; Mahoney, 1977), at levels only slightly better than chance ( Herron, 2012; Smith, 2006). Studies suggest decisions on rejection or acceptance are similarly inconsistent. For example, Peters and Ceci’s classic study found that eight out of twelve papers were rejected for methodological flaws when resubmitted to the same journals in which they had already been published ( Peters & Ceci, 1982). This inconsistency is mirrored in peer review’s inability to prevent errors and fraud from entering the scientific literature. Reviewers often fail to detect major methodological failings ( Schroter *et al.*, 2004), with eminent journals (whose higher rejection rates might suggest more stringent peer review processes) seeming to perform no better than others ( Fang *et al.*, 2012). Indeed, Fang and Casadevall found that the frequency of retraction is strongly correlated with the journal impact factor ( Fang & Casadevall, 2011). Whatever the cause, recent sharp rises in the number of retracted scientific publications ( Steen *et al.*, 2013) testify that peer review sometimes fails in its role as the gatekeeper of science, allowing errors and fraudulent material to enter the literature. At an editorial level, peer review’s other role, of guiding decisions that should in theory filter the best work into the best journals, also seems to be found wanting. Many articles in top journals remain poorly cited, while many of the most highly-cited articles in their fields are published in lower-tier journals ( Jubb, 2016).
- **Delay and expense:** The period from submission to publication at many journals can often exceed one year, with much of this time taken up by peer review. This delay slows down the availability of results for further research and professional exploitation. The work undertaken in this period is also expensive, with the global costs of reviewers’ time estimated at £1.9bn in 2008 ( Research Information Network [RIN], 2008), a figure which does not take into account the coordinating costs of publishers, or the time authors spend revising and resubmitting manuscripts ( Jubb, 2016). These costs are greatly exacerbated by the current system in which peer review is managed by each journal, such that the same manuscript may be peer reviewed many times over as it is successively rejected and resubmitted until it finds acceptance. It could be argued that these issues relate more to editorial process than peer review *per se*. However, as we shall see, various new publishing models which encompass innovations in peer review (including open peer review), have the potential to address such issues.
- **Lack of accountability and risks of subversion:** The “black-box” nature of traditional peer review gives reviewers, editors and even authors a lot of power to potentially subvert the process. At the editorial level, lack of transparency means that editors can unilaterally reject submissions or shape review outcomes by selecting reviewers based on their known preference for or aversion to certain theories and methods ( Travis & Collins, 1991). Reviewers, shielded by anonymity, may act unethically in their own interests by concealing conflicts of interest. Smith, an experienced editor, for example, reports reviewers stealing ideas and passing them off as their own, or intentional blocking or delaying publication of competitors’ ideas through harsh reviews ( Smith, 2006). Equally, they may simply favour their friends and target their enemies. Authors, meanwhile, can manipulate the system by writing reviews of their own work via fake or stolen identities ( Kaplan, 2015).
- **Social and publication biases:** Although often idealized as impartial, objective assessors, in reality studies suggest that peer reviewers may be subject to social biases on the grounds of gender ( Budden *et al.*, 2008; Lloyd, 1990; Tregenza, 2002), nationality ( Daniel, 1993; Ernst & Kienbacher, 1991; Link, 1998), institutional affiliation ( Dall’Aglio, 2006; Gillespie *et al.*, 1985; Peters & Ceci, 1982), language ( Cronin, 2009; Ross *et al.*, 2006; Tregenza, 2002) and discipline ( Travis & Collins, 1991). Other studies suggest so-called “publication bias”, where prejudices against speciﬁc categories of works shape what is published. Publication bias can take many forms. First is a preference for complexity over simplicity in methodology (even if inappropriate, c.f. Travis & Collins, 1991) and language ( Armstrong, 1997). Next, “confirmatory bias” is theorized to lead to conservatism, biasing reviewers against innovative methods or results contrary to dominant theoretical perspectives ( Chubin & Hackett, 1990; Garcia *et al.*, 2016; Mahoney, 1977). Finally, factors like the pursuit of “impact” and “excellence” ( Moore *et al.*, 2017) mean that editors and reviewers seem primed to prefer positive results over negative or neutral ones ( Bardy, 1998; Dickersin *et al.*, 1992; Fanelli, 2010; Ioannidis, 1998), and to disfavour replication studies ( Campanario, 1998; Kerr *et al.*, 1977).
- **Lack of incentives**: Traditional peer review provides little in the way of incentives for reviewers, whose work is almost exclusively unpaid and whose anonymous contributions cannot be recognised and hence rewarded ( Armstrong, 1997; Ware, 2008).
- **Wastefulness:** Reviewer comments often add context or point to areas for future work. Reviewer disagreements can expose areas of tension in a theory or argument. The behind-the-scenes discussions of reviewers and authors can also guide younger researchers in learning review processes. Readers may find such information helpful and yet at present, this potentially valuable additional information is wasted.

In response to these criticisms, a wide variety of changes to peer review have been suggested (see the extensive overviews in Tennant *et al.*, 2017; Walker & Rocha da Silva, 2015). Amongst these innovations, many have been labelled as “open peer review” at one time or another. As we shall see, these innovations labelled as OPR in fact encompass a wide variety of discrete ways in which peer review can be “opened up”. Each of these distinct traits are theorized to address one or more of the shortcomings listed above, but no trait is claimed to address all of them and sometimes their aims may be in conflict. These points will be addressed fully in the discussion section.

### 2. The contested meaning of open peer review

The diversity of the definitions provided for open peer review can be seen by examining just two examples. The first one is, to my knowledge, the first recorded use of the phrase “open peer review”:

“[A]n open reviewing system would be preferable. It would be more equitable and more efficient. Knowing that they would have to defend their views before their peers should provide referees with the motivation to do a good job. Also, as a side benefit, referees would be recognized for the work they had done (at least for those papers that were published). Open peer review would also improve communication. Referees and authors could discuss difficult issues to find ways to improve a paper, rather than dismissing it. Frequently, the review itself provides useful information. Should not these contributions be shared? Interested readers should have access to the reviews of the published papers.” ( Armstrong, 1982)

“[O]pen review makes submissions OA [open access], before or after some prepublication review, and invites community comments. Some open-review journals will use those comments to decide whether to accept the article for formal publication, and others will already have accepted the article and use the community comments to complement or carry forward the quality evaluation started by the journal. ” ( Suber, 2012)

Within just these two examples, there are already a multitude of factors at play, including the removal of anonymity, the publishing of review reports, interaction between participants, crowdsourcing of reviews, and making manuscripts public pre-review, amongst others. But each of these are distinct factors, presenting separate strategies for openness and targeting differing problems. For example, disclosure of identities aims usually at increasing accountability and minimizing bias, c.f. “referees should be more highly motivated to do a competent and fair review if they may have to defend their views to the authors and if they will be identified with the published papers” ( Armstrong, 1982). Publication of reports, on the other hand, also tackles problems of incentive (reviewers can get credit for their work) and wastefulness (reports can be consulted by readers). Moreover, these factors need not necessarily be linked, which is to say that they can be employed separately: identities can be disclosed without reports being published, and reports published with reviewer names withheld, for example.

This diversity has led many authors to acknowledge the essential ambiguity of the term “open peer review” ( Hames, 2014; Sandewall, 2012; Ware, 2011). The major attempt thus far to bring coherence to this confusing landscape of competing and overlapping definitions, is Emily Ford’s paper “Defining and Characterizing Open Peer Review: A Review of the Literature” ( Ford, 2013). Ford examined thirty-five articles to produce a schema of eight “common characteristics” of OPR: signed review, disclosed review, editor-mediated review, transparent review, crowdsourced review, prepublication review, synchronous review, and post-publication review. Unfortunately, however, Ford’s paper fails to offer a definitive definition of OPR, since despite distinguishing eight “common characteristics” of OPR, Ford nevertheless tries to reduce it to merely one: open identities: “Despite the differing definitions and implementations of open peer review discussed in the literature, its general treatment suggests that the process incorporates disclosure of authors’ and reviewers’ identities at some point during an article’s review and publication” (p. 314). Summing up her argument elsewhere, she says: “my previous definition … broadly understands OPR as any scholarly review mechanism providing disclosure of author and referee identities to one another” ( Ford, 2015). But the other elements of her schema do not reduce to this one factor. Many definitions do not include open identities at all. This hence means that although Ford claims to have identified several features of OPR, she in fact is asserting that there is only one *defining* factor (open identity), which leaves us where we started. Ford’s schema is also problematic elsewhere: it lists “editor-mediated review” and “pre-publication review” as distinguishing characteristics, despite these being common traits of traditional peer review; it includes questionable elements such as the purely “theoretical” “synchronous review”; and some of its characteristics do not seem to be “base elements”, but complexes of other traits – for example, the definition of “transparent review” incorporates other characteristics such as open identities (which Ford terms “signed review”) and open reports (“disclosed review”).

## Method: A systematic review of previous definitions

To resolve this ambiguity, I performed a review of the literature for articles discussing “open review” or “open peer review”, extracting a corpus of 122 definitions of OPR. I first searched Web of Science (WoS) for “TOPIC: (”open review" OR “open peer review”)”, with no limitation on date of publication, yielding a total of 137 results (searched on 12th July 2016). These records were then each individually examined for relevance and a total of 57 were excluded. 21 results (all BioMed Central publications) had been through an OPR process (which was mentioned in the abstract) but did not themselves touch on the subject of OPR; 12 results used the phrase “open review” to refer to a literature review with a flexible methodology; 12 results were for the review of objects classed “out of scope” (i.e. academic articles, books, conference submissions, data – examples included guidelines for clinical or therapeutic techniques, standardized terminologies, patent applications, and court judgements); 7 results were not in the English language; and 5 results were duplicate entries in WoS. This left a total of 80 relevant articles which mentioned either “open peer review” or “open review”.

The same search terms were applied to find sources in other academic databases (Google Scholar, PubMed, ScienceDirect, JSTOR and Project Muse). In addition, the first 10 pages of search results for these terms in Google and Google Books (search conducted 18 ^th^ July 2016) were examined to find references in “grey literature” (blogs, reports, white papers) and books respectively. Finally, the author examined the reference sections of identified publications, especially bibliographies and literature reviews, to find further references. Duplicate results were discarded and the above exclusion criteria applied to add a further 42 definitions to the corpus. The dataset is available online ( Ross-Hellauer, 2017, http://doi.org/10.5281/zenodo.438024).

Each source was then individually examined for its definition of OPR. Where no explicit definition (e.g. “OPR is …”) was given, implicit definitions were gathered from contextual statements. For instance, “reviewers can notify the editors if they want to opt-out of the open review system and stay anonymous” ( Janowicz & Hitzler, 2012) is taken to endorse a definition of OPR as incorporating open identities. In a few cases, sources defined OPR in relation to the systems of specific publishers (e.g., F1000Research, BioMed Central and Nature), and so were taken to implicitly endorse those systems as definitive of OPR.

In searching only for the terms “open review” and “open peer review”, the study explicitly limits itself only to that literature which uses these terms. It is hence important to note that it is likely that other studies have described or proposed innovations to peer review which have aims similar to those identified by this study. However, if they have not explicitly used the label “open review” or “open peer review” in conjunction with these systems, those studies would necessarily fall outside of scope. For example, “post-publication peer review” (PPPR) is clearly a concept closely-related to OPR, but unless sources explicitly equate the two, sources discussing PPPR are not included in this review. It is acknowledged that this focus on the distinct usages of the term OPR, rather than on all sources which touch on the various aims and ideas which underlie such systems, limits the scope of this study.

## Results

**Figure 1.**
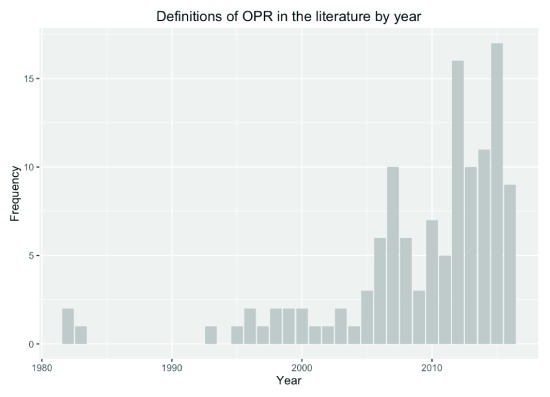
Definitions of OPR in the literature by year.

**Figure 2.**
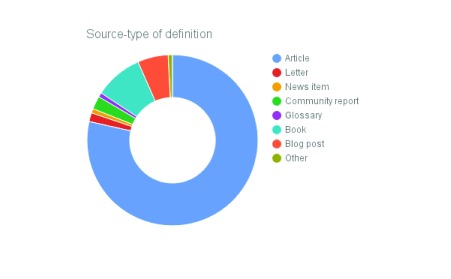
Breakdown of OPR definitions by source.

**Figure 3.**
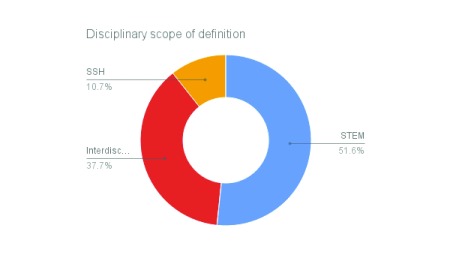
Breakdown of OPR definitions by disciplinary scope.

**Figure 4.**
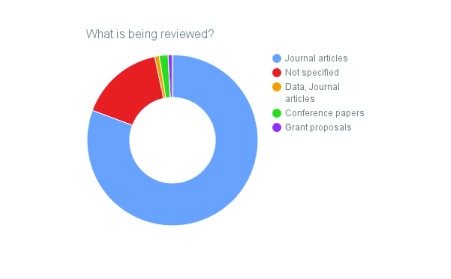
Breakdown of OPR definitions by type of material being reviewed.

The number of definitions of OPR over time show a clear upward trend, with the most definitions in a single year coming in 2015. The distribution shows that except for some outlying definitions in the early 1980s, the phrase “open peer review” did not really enter academic discussion until the early 1990s. At that time, the phrase seems to have been used largely to refer to non-blinded review (i.e. open identities). There is then a big upswing from the early-mid 2000s onwards, which perhaps correlates with the rise of the rise of the openness agenda (especially open access, but also open data and open science more generally) over that period ( Figure 1). Most of the definitions, 77.9% (n=95), come from peer-reviewed journal articles, with the second largest sources being books and blog posts. Other sources include letters to journals, news items, community reports and glossaries ( Figure 2). As shown in Figure 3, the majority of definitions (51.6%) were identified to be primarily concerned with peer-review of Science, Technology, Engineering and Medicine (STEM) subject material, while 10.7% targeted material from Social Sciences and Humanities (SSH) material. The remainder (37.7%) were interdisciplinary. Meanwhile, regarding the target of the OPR mentioned in these articles ( Figure 4), most were referring to peer review of journal articles (80.7%), with 16% not specifying a target (16%), and a small number of articles also referring to review of data, conference papers and grant proposals.

Sixty-eight percent (n=83) of the 122 definitions identified were explicitly stated, 37.7% (n=46) implicitly stated, and 5.7% (n=7) contained both explicit and implicit information.

The extracted definitions were examined and classified against an iteratively constructed taxonomy of OPR traits. Nickerson *et al.* (2013) advise that the development of a taxonomy should begin by identifying the appropriate meta-characteristic – in this case distinct individual innovations to the traditional peer review system. An iterative approach then followed, in which dimensions given in the literature were applied to the corpus of definitions and gaps/overlaps in the OPR taxonomy identified. Based on this, new traits or distinctions were introduced so that in the end, a schema of seven OPR traits was produced:

- **Open identities:** Authors and reviewers are aware of each other’s identity
- **Open reports:** Review reports are published alongside the relevant article.
- **Open participation:** The wider community are able to contribute to the review process.
- **Open interaction:** Direct reciprocal discussion between author(s) and reviewers, and/or between reviewers, is allowed and encouraged.
- **Open pre-review manuscripts:** Manuscripts are made immediately available (e.g., via pre-print servers like arXiv) in advance of any formal peer review procedures.
- **Open final-version commenting:** Review or commenting on final “version of record” publications.
- **Open platforms (“decoupled review”):** Review is facilitated by a different organizational entity than the venue of publication.

**Figure 5.**
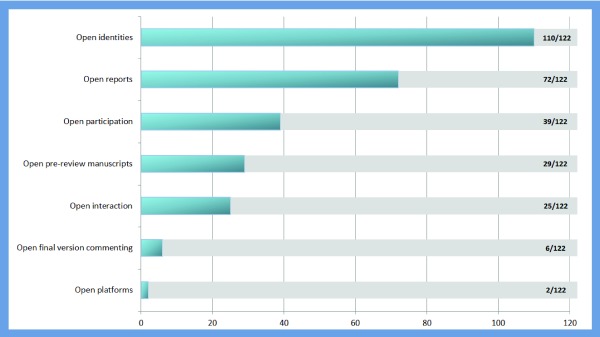
Distribution of OPR traits amongst definitions.

The core traits are easily identified, with just three covering more than 99% of all definitions: Open identities combined with open reports cover 116 (95.1%) of all records. Adding open participations leads to a coverage of 121 (99.2%) records overall. As seen in Figure 5, open identities is by far the most prevalent trait, present in 90.1% (n=110) of definitions. Open reports is also present in the majority of definitions (59.0%, n=72), while open participation is part of around a third. Open pre-review manuscripts (23.8%, n=29) and open interaction (20.5%, n=25) are also a fairly prevalent part of definitions. The outliers are open final version commenting (4.9%) and open platforms (1.6%).

**Figure 6.**
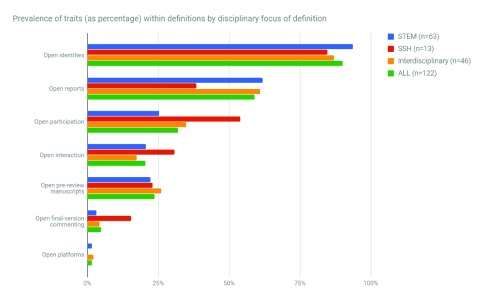
Prevalence of traits (as percentage) within definitions by disciplinary focus of definition.

If we break down these traits by the disciplinary-focus of the definition source, we observe some interesting differences between STEM- and SSH-focused sources ( Figure 6). Of those sources whose definitions were identified to be primarily concerned with peer-review of SSH-subject material, we observe that in comparison to STEM, there is less emphasis on open identities (present in 84.6% of SSH-focused definitions compared to 93.7% of STEM-focused definitions) and open reports (38.5% SSH vs. 61.9% STEM). Three traits were much more likely to be included in SSH definitions of OPR, however: open participation (53.85% SSH vs. 25.4% STEM), open interaction (30.8% SSH vs. 20.6% STEM), and open final-version commenting (15.4% SSH vs. 3.2%STEM). The other traits, open pre-review manuscripts and open platforms, were similar across both groups. Although these differences seem to hint at a slightly different understanding of OPR between the disciplines, we should be careful in generalizing too strongly here. Firstly because splitting scholarship into these two broad groups risks levelling the wealth of disciplinary-specificity within these categories. Secondly, because the number of SSH-specific sources (13) was small.

**Figure 7.**
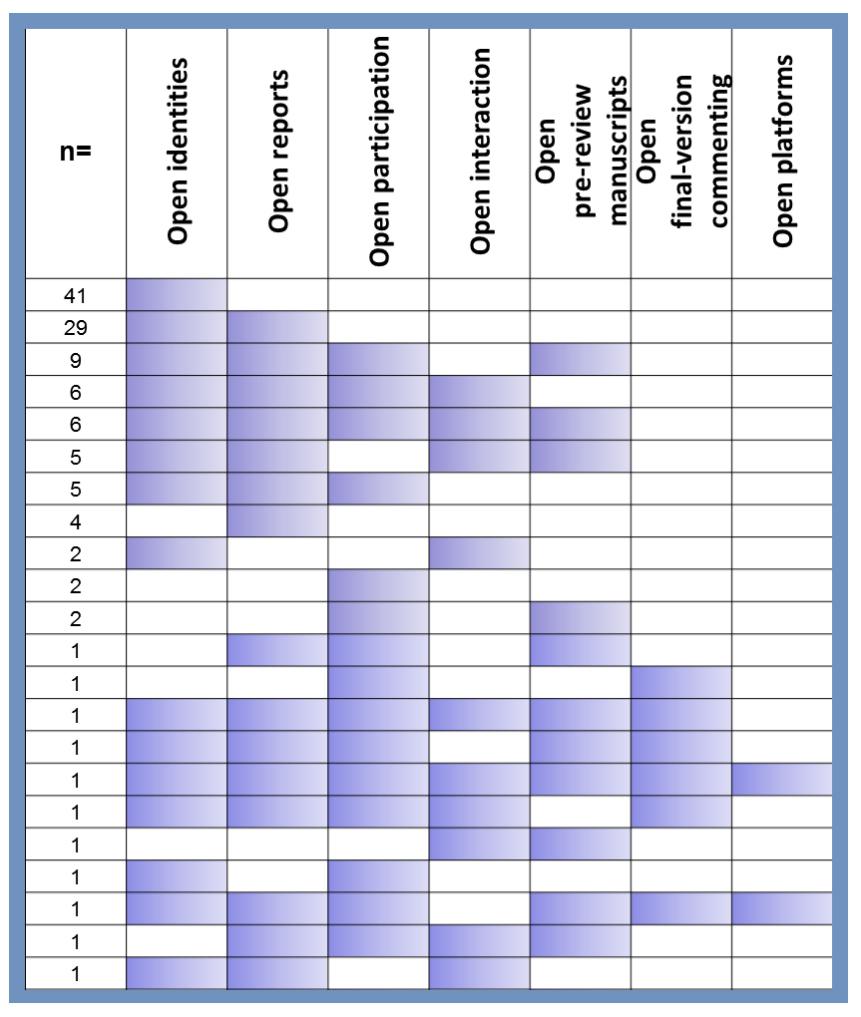
Unique configurations of OPR traits within definitions.

The various ways these traits are configured within definitions can be seen in Figure 7. Quantifying definitions in this way allows us to accurately portray exactly how ambiguously the phrase “open peer review” has been used thus far, for the literature offers a total of 22 distinct configurations of seven traits, effectively meaning that there are 22 different definitions of OPR in the literature examined here.

The distribution of traits shows two very popular configurations and a variety of rarer ones, with the most popular configuration (open identities) accounting for one third (33.6%, n=41) and the second-most popular configuration (open identities, open reports) accounting for almost a quarter (23.8%, n=29) of all definitions. There then follows a “long-tail” of less-frequently found configurations, with more than half of all configurations being unique to a single definition.

## Discussion: The traits of open peer review

I next offer a detailed analysis of each of these traits, detailing the issues they aim to resolve and the evidence to support their effectiveness.

### Open identities

Open identity peer review, also known as signed peer review ( Ford, 2013; Nobarany & Booth, 2015) and “unblinded review” ( Monsen & Van Horn, 2007), is review where authors and reviewers are aware of each other’s identities. Traditional peer review operates as either “single-blind”, where authors do not know reviewers’ identities, or “double-blind”, where both authors and reviewers remain anonymous. Double-blind reviewing is more common in the Arts, Humanities and Social Sciences than it is in STEM (science, technology, engineering and medicine) subjects, but in all areas single-blind review is by far the most common model ( Walker & Rocha da Silva, 2015). A main reason for maintaining author anonymity is that it is assumed to tackle possible publication biases against authors with traditionally feminine names, from less prestigious institutions or non-English speaking regions ( Budden *et al.*, 2008; Ross *et al.*, 2006). Reviewer anonymity, meanwhile, is presumed to protect reviewers from undue influence, allowing them to give candid feedback without fear of possible reprisals from aggrieved authors. Various studies have failed to show that such measures increase review quality, however ( Fisher *et al.*, 1994; Godlee *et al.*, 1998; Justice *et al.*, 1998; McNutt *et al.*, 1990; van Rooyen *et al.*, 1999). As Godlee and her colleagues have said, “Neither blinding reviewers to the authors and origin of the paper nor requiring them to sign their reports had any effect on rate of detection of errors. Such measures are unlikely to improve the quality of peer review reports” ( Godlee *et al.*, 1998). Moreover, factors such as close disciplinary communities and internet search capabilities, mean that author anonymity is only partially effective, with reviewers shown to be able to identify authors in between 26 and 46 percent of cases ( Fisher *et al.*, 1994; Godlee *et al.*, 1998).

Proponents of open identity peer review argue that it will enhance accountability, further enable credit for peer reviewers, and simply make the system fairer: “most importantly, it seems unjust that authors should be “judged” by reviewers hiding behind anonymity” ( van Rooyen *et al.*, 1999). Open identity peer review is argued, moreover, to potentially increase review quality, as it is theorised that reviewers will be more highly motivated and invest more care in their reviews if their names are attached to them. Finally, a reviewer for this paper advises that “proponents of open identity review in medicine would also point out that it makes conflicts of interest much more apparent and subject to scrutiny” ( Bloom, 2017). Opponents counter this by arguing that signing will lead to poorer reviews, as reviewers temper their true opinions to avoid causing offence. To date, studies have failed to show any great effect in either direction ( McNutt *et al.*, 1990; van Rooyen *et al.*, 1999; van Rooyen *et al.*, 2010). However, since these studies derive from only one disciplinary area (medicine), the results cannot be taken as representative and hence further research is undoubtedly required.

### Open reports

Open reports peer review is where review reports (either full reports or summaries) are published alongside the relevant article. Often, although not in all cases (e.g., EMBO reports, http://embor.embopress.org), review names are published alongside the reports. The main benefits of this measure lie in making currently invisible but potentially useful scholarly information available for re-use. There is increased transparency and accountability that comes with being able to examine normally behind-the-scenes discussions and processes of improvement and assessment, and a potential to further incentivize peer reviewers by making their peer review work a more visible part of their scholarly activities (thus enabling reputational credit).

Reviewing is hard work. Research Information Network reported in 2008 that a single peer review takes an average of four hours, at an estimated total annual global cost of around £1.9 billion ( Research Information Network, 2008). Once an article is published, however, these reviews usually serve no further purpose than to reside in publisher’s long-term archives. Yet those reviews contain information that remains potentially relevant and useful in the here-and-now. Often, works are accepted despite the lingering reservations of reviewers. Published reports can enable readers to consider these criticisms themselves, and “have a chance to examine and appraise this process of “creative disagreement” and form their own opinions” ( Peters & Ceci, 1982). Making reviews public in this way also adds another layer of quality assurance, as the reviews are open to the scrutiny of the wider scientific community. It could also increase review quality, as the thought of their words being made publicly available could motivate reviewers to be more thorough in their review activities. Moreover, publishing reports also aims at raising the recognition and reward of the work of peer reviewers. Adding review activities to the reviewer’s professional record is common practice; author identification systems currently also add mechanisms to host such information (e.g. via ORCID) ( Hanson *et al.*, 2016). Finally, open reports give young researchers a guide (to tone, length, the formulation of criticisms) to help them as they begin to do peer review themselves.

The evidence-base against which to judge such arguments is not great enough to enable strong conclusions, however. Van Rooyen and her colleagues found that open reports correlate with higher refusal rates amongst potential reviewers, as well as an increase in time taken to write review but no concomitant effect on review quality ( van Rooyen *et al.*, 2010). Nicholson and Alperin’s small survey, however, found generally positive attitudes: “researchers … believe that open review would generally improve reviews, and that peer reviews should count for career advancement” ( Nicholson & Alperin, 2016).

### Open participation

Open participation peer review, also known as “crowdsourced peer review” ( Ford, 2013; Ford, 2015), “community/public review” ( Walker & Rocha da Silva, 2015) and “public peer review” ( Bornmann *et al.*, 2012), allows the wider community to contribute to the review process. Whereas in traditional peer review editors identify and invite specific parties (peers) to review, open participation processes invite interested members of the scholarly community to participate in the review process, either by contributing full, structured reviews or shorter comments. According to Fitzpatrick & Santo (2012), the rationale for opening up the pool of reviewers in this way is that “fields can often become self-replicating, as they limit the input that more horizontally-organized peer groups – such as scholars from related disciplines and interdisciplines, and even members of more broadly understood publics – might play in the development of scholarly thought” ( Fitzpatrick & Santo, 2012).

In practice, it may be that comments are open to anybody (anonymous or registered), or some credentials might first be required (e.g., Science Open requires an ORCID profile with at least five published articles). Open participation is often used as a complement to a parallel process of solicited peer review. It aims to resolve possible conflicts associated with editorial selection of reviewers (e.g. biases, closed-networks, elitism) and possibly improve the reliability of peer review by increasing the number of reviewers ( Bornmann *et al.*, 2012). Reviewers can come from the wider research community, as well as those traditionally under-represented in scientific assessment, including representatives from industry or members of special-interest groups, for example patients in the case of medical journals ( Ware, 2011). This has the potential to open the pool of reviewers beyond those identified by editors to include all potentially interested reviewers (including those from outside academia), and hence increase the number of reviewers for each publication (though in practice this is unlikely). Evidence suggests this practice could help increase the accuracy of peer review. For example, Herron (2012) produced a mathematical model of the peer review process which showed that “the accuracy of public reader-reviewers can surpass that of a small group of expert reviewers if the group of public reviewers is of sufﬁcient size”, although only if the numbers of reader-reviewers exceeded 50.

Criticisms of open participation routinely focus on questions about reviewers’ qualifications to comment and the incentives for doing so. Given that disciplines are subject to increasingly narrow specialization, especially in the sciences ( Casadevall & Fang, 2014), it can be objected that those who lack intimate knowledge of the particular methods and objects of that field will literarily be unable to properly evaluate findings. As Stevan Harnad has said: “it is not clear whether the self-appointed commentators will be qualified specialists (or how that is to be ascertained). The expert population in any given speciality is a scarce resource, already overharvested by classical peer review, so one wonders who would have the time or inclination to add journeyman commentary services to this load on their own initiative” ( Harnad, 2000). Here, we might reflect on whether this is one reason why open participation seems to be a more central part of conceptions of OPR in the social science and humanities than in STEM subjects. As we saw above, open participation is actually the second most popular trait in definitions stemming from sources with an SSH-focus, appearing in more than half of those definitions, as compared to just a quarter of definitions that focused specifically on STEM subjects (although, again, we must remind ourselves that the small number of SSH definitions means we should not draw overly-strong conclusions based on this finding). As Fitzpatrick and Santo argue, in the humanities, peer review “often focuses on originality, creativity, depth and cogency of argument, and the ability to develop and communicate new connections across and additions to existing texts and ideas”. This is contrasted to the sciences, where peer review is more concretely focused on “verification of results or validation of methodologies” ( Fitzpatrick & Santo, 2012). Assessment of narrative cogency and the interconnection of ideas are more transferable across domains than are knowledge of discipline-specific methods and tools. To be sure, both play a role in all scholarship, but since the former play a larger role in SSH, this may be a motivating factor in increased interest in open participation in those disciplines.

Another issue for open participation is that difficulties have been reported in motivating self-selecting commentators to take part and deliver useful critique. *Nature*, for example, ran an experiment from June to December 2006 inviting submitting authors to take part in an experiment where open participation would be used as a complement to a parallel process of solicited peer reviews. Nature judged the trial to have been unsuccessful due to the small number of authors wishing to take part (just 5% of submitting authors), the small number of overall comments (almost half of articles received no comments) and the insubstantial nature of most of the comments that were received ( Fitzpatrick, 2011). At the open access journal *Atmospheric Chemistry and Physics* (ACP), which publishes pre-review discussion papers for community comments, only about one in ﬁve papers is commented upon ( **Pöschl, 2012**). **Bornmann *et al.* (2012)** conducted a comparative content analysis of the ACP’s community comments and formal referee reviews and concluded that the latter – tending to focus more on formal qualities, conclusions and potential impact – better supported the selection and improvement of manuscripts. This all suggests that although open participation might be a worthwhile complement to traditional, invited peer review, it is unlikely to be able to fully replace it.

### Open interaction

Open interaction peer review allows and encourages direct reciprocal discussion between reviewers, and/or between author(s) and reviewers. In traditional peer review, reviewers and authors correspond only with editors. Reviewers have no contact with other reviewers, and authors usually have no opportunity to directly question or respond to reviewers’ comments. Allowing interaction amongst reviewers or between authors and reviewers, or between reviewers themselves, is another way to “open up” the review process, enabling editors and reviewers to work with authors to improve their manuscript. The motivation for doing so, according to ( Armstrong, 1982), is to “improve communication. Referees and authors could discuss difficult issues to find ways to improve a paper, rather than dismissing it”. In the words of Kathleen Fitzpatrick (2012), such interaction can foster “a conversational, collaborative discourse that not only harkens back to the humanities’ long investment in critical dialogue as essential to intellectual labor, but also models a forward-looking approach to scholarly production in a networked era.”

Some journals enable pre-publication interaction between reviewers as standard ( Hames, 2014). The *EMBO Journal*, for example, enables “cross-peer review,” where referees are “invited to comment on each other’s reports, before the editor makes a decision, ensuring a balanced review process” ( EMBO Journal, 2016). At *eLife*, reviewers and editor engage in an “online consultation session” where they come to a mutual decision before the editor compiles a single peer review summary letter for the author to give them a single, non-contradictory roadmap for revisions ( Schekman *et al.*, 2013). The publisher Frontiers has gone a step further, including an interactive collaboration stage that “unites authors, reviewers and the Associate Editor – and if need be the Specialty Chief Editor – in a direct online dialogue, enabling quick iterations and facilitating consensus” ( Frontiers, 2016).

Perhaps even more so than other areas studied here, evidence to judge the effectiveness of interactive review is scarce. Based on anecdotal evidence, Walker & Rocha da Silva (2015) advise that “[r]eports from participants are generally but not universally positive”. To the knowledge of the author, the only experimental study that has specifically examined interaction among reviewers or between reviewers and authors is that of Jeffrey Leek and his colleagues, who performed a laboratory study of open and closed peer review based on an online game and found that “improved cooperation does in fact lead to improved reviewing accuracy. These results suggest that in this era of increasing competition for publication and grants, cooperation is vital for accurate evaluation of scientific research” ( Leek *et al.*, 2011). Such results are encouraging, but hardly conclusive. Hence, there remains much scope for further research to determine the impact of cooperation on the efficacy and cost of the review process.

### Open pre-review manuscripts

Open pre-review manuscripts are manuscripts that are immediately openly accessible (via the internet) in advance, or in synchrony with, any formal peer review procedures. Subject-specific “preprint servers” like arXiv.org and bioRxiv.org, institutional repositories, catch-all repositories like Zenodo or Figshare and some publisher-hosted repositories (like *PeerJ Preprints*) allow authors to short-cut the traditional publication process and make their manuscripts immediately available to everyone. This can be used as a complement to a more traditional publication process, with comments invited on preprints and then incorporated into redrafting as the manuscript goes through traditional peer review with a journal. Alternatively, services which overlay peer-review functionalities on repositories can produce functional publication platforms at reduced cost ( Boldt, 2011; Perakakis *et al.*, 2010). The mathematics journal *Discrete Analysis*, for example, is an overlay journal whose primary content is hosted on arXiv ( Day, 2015). The recently released Open Peer Review Module for repositories, developed by Open Scholar in association with OpenAIRE, is an open source software plug-in which adds overlay peer review functionalities to repositories using the DSpace software ( OpenAIRE, 2016). Another innovative model along these lines is that of *ScienceOpen*, which ingests articles metadata from preprint servers and contextualizes them by adding altmetrics and other relational information, before offering authors peer review.

In other cases, manuscripts are submitted to publishers in the usual way but made immediately available online (usually following some rapid preliminary review or “sanity check”) before the start of the peer review process. This approach was pioneered with the 1997 launch of the online journal *Electronic Transactions in Artificial Intelligence* (ETAI), where a two-stage review process was used. First, manuscripts were made available online for interactive community discussion, before later being subject to standard anonymous peer review. The journal stopped publishing in 2002 ( Sandewall, 2012). *Atmospheric Chemistry and Physics* uses a similar system of multi-stage peer review, with manuscripts being made immediately available as “discussion papers” for community comments and peer review ( Pöschl, 2012). Other prominent examples are *F1000Research* and the *Semantic Web Journal*.

The benefits to be gained from open pre-review manuscripts is that researchers can assert their priority in reporting findings – they needn’t wait for the sometimes seemingly endless peer review and publishing process, during which they might fear being scooped. Moreover, getting research out earlier increases its visibility, enables open participation in peer review (where commentary is open to all), and perhaps even, according to ( Pöschl, 2012), increases the quality of initial manuscript submissions. Finally, making manuscripts openly available in advance of review allows comments to be posted as they are received, either from invited reviewers or the wider community, and enabling readers to follow the process of peer-review in real-time.

### Open final-version commenting

Open final-version commenting is review or commenting on final “version of record” publications. If the purpose of peer review is to assist in the selection and improvement of manuscripts for publication, then it seems illogical to suggest that peer review can continue once the final version-of-record is made public. Nonetheless, in a literal sense, even the declared fixed version-of-record continues to undergo a process of improvement (occasionally) and selection (perpetually).

The internet has hugely expanded the range of effective action available for readers to offer their feedback on scholarly works. Where before only formal routes like the letters to the journal or commentary articles offered readers a voice, now a multitude of channels exist. Journals are increasingly offering their own commentary sections. Walker & Rocha da Silva (2015) found that of 53 publishing venues reviewed, 24 provided facilities to enable user-comments on published articles – although these were typically not heavily used. Researchers seem to see the worth of such functionalities, with almost half of respondents to a 2009 survey believing supplementing peer review with some form of post-publication commentary to be beneficial ( Mulligan *et al.*, 2013). But users can “publish” their thoughts anywhere on the Web – via academic social networks like *Mendeley*, *ResearchGate* and *Academia*. *edu*, via *Twitter*, or on their own blogs. In this sense, peer review can be decoupled not only from the journal, but also from any particular platform. The reputation of a piece of work is continuously evolving as long as it remains the subject of discussion. Thus, considering final-version commenting to be an active part of an ongoing, perpetual process peer review in a wider sense hence might encourage an adjustment in our conception of the nature of peer review, away from seeing it as a distinct process that leads to publication, and Improvements based on feedback happen most obviously in the case of so-called ‘living’ publications, like the *Living Reviews* group of three disciplinary journals in the fields of relativity, solar physics and computational astrophysics, publishing invited review articles which allow authors to regularly update their articles to incorporate the latest developments in the field. Here, even where the published version is anticipated to be the final version, it remains open to future retraction or correction. Such changes are often fueled by social media, as in the 2010 case of #arseniclife, where social media critique over flaws in the methodology of a paper claiming to show a bacterium capable of growing on arsenic resulted in refutations being published in Science. The *Retraction Watch* blog is dedicated to publicizing such cases.

An important platform in this regard has been *Pubpeer* which proclaims itself a “post-publication peer review platform”. When its users swarmed to critique a *Nature* paper on STAP (Stimulus-Triggered Acquisition of Pluripotency) cells, PubPeer argued that its “post-publication peer review easily outperformed even the most careful reviewing in the best journal. The papers’ comment threads on PubPeer have attracted some 40000 viewers. It’s hardly surprising they caught issues that three overworked referees and a couple of editors did not. Science is now able to self-correct instantly. Post-publication peer review is here to stay” ( PubPeer, 2014).

### Open platforms (“decoupled review”)

Open platforms peer review is review facilitated by a different organizational entity than the venue of publication. Recent years have seen the emergence of a group of dedicated platforms which aim to augment the traditional publishing ecosystem by de-coupling review functionalities from journals. Services like RUBRIQ and Peerage of Science offer “portable” or “independent” peer review. A similar service, Axios Review, operated from 2013 to 2017. Each platform invites authors to submit manuscripts directly to them, then organises review amongst their own community of reviewers and returns review reports. In the case of RUBRIQ and Peerage of Science, participating journals then have access to these scores and manuscripts and so can contact authors with a publishing offer or to suggest submission. Axios meanwhile, directly forwarded the manuscript, along with reviews and reviewer identities, to the author’s preferred target journal. The models vary in their details – RUBRIQ, for example, pays its reviewers, whereas Axios operated on a community model where reviewers earned discounts on having their own work reviewed – but all aim in their ways to reduce inefficiencies in the publication process, especially the problem of duplication of effort. Whereas in traditional peer review, a manuscript could undergo peer review at several journals, as it is submitted and rejected, then submitted elsewhere, such services need just one set of reviews which can be carried over to multiple journals until a manuscript finds a home (hence “portable” review).

Other decoupled platforms aim at solving different problems. Publons seeks to address the problem of incentive in peer review by turning peer review into measurable research outputs. Publons collects information about peer review from reviewers and publishers to produce reviewer profiles which detail verified peer review contributions that researchers can add to their CVs. Overlay journals like *Discrete Mathematics*, discussed above, are another example of open platforms. Peter Suber (quoted in Cassella & Calvi, 2010) defines the overlay journal as “An open-access journal that takes submissions from the preprints deposited at an archive (perhaps at the author’s initiative), and subjects them to peer review…. Because an overlay journal doesn’t have its own apparatus for disseminating accepted papers, but uses the pre-existing system of interoperable archives, it is a minimalist journal that only performs peer review.” Finally, there are the many venues through which readers can now comment on already-published works (see also “open final-version commenting” above), including blogs and social networking sites, as well as dedicated platforms such as PubPeer.

### Which problems with traditional peer do the various OPR traits address?

I began by sketching out various problems with traditional peer review and advised that OPR, in its various incarnations, has been proposed as a solution to many of these problems, but that no individual trait addresses all of these problems, and that sometimes their aims may be in conflict. Which traits address which of the problems identified above? Which might actually exacerbate them? Based on the foregoing, I here present this summary:

- **Unreliability and inconsistency:** *Open identities* and *open reports* are theorized to lead to better reviews, as the thought of having their name publicly connected to a work or seeing their review published encourages reviewers to be more thorough. There is at present too little evidence to judge if this is actually so, however. *Open participation* and *open final-version commenting* are theorized to possibly improve the reliability of peer review by increasing the number of potential reviewers, especially from different disciplinary backgrounds. In practice, open participation struggles to attract reviewers in most cases and thus is probably not a sustainable replacement for invited peer review, although it is perhaps a worthwhile supplement to it. Some evidence suggests that *open interaction* between reviewers and authors could lead to improved reviewing accuracy.
- **Delay and expense:** *Open pre-review manuscripts* sharply reduce the time before research is first publicly available and may increase the overall quality of initial submissions. *Open platforms* can help overcome the “waterfall” problem, where individual articles go through multiple cycles of review and rejection at different journals. In principle, *open participation* could reduce the need for editorial mediation in finding reviewers, but in practice any reduction of costs is questionable, as open participation can fail to attract reviewers and in any case, editorial mediation will continue to be necessary to facilitate discussion and arbitrate disputes. *Open identities* and *open reports* might actually exacerbate problems of delay and expense, as it seems invited reviewers are currently less inclined to review under such circumstances. Finally, *open interaction* – by necessitating more back and forth between reviewers and authors, and more editorial mediation – might lead to longer reviewing times.
- **Lack of accountability and risks of subversion:** *Open identities* and *reports* can increase accountability through increased transparency and by making any conflicts of interest more immediately apparent to authors and future readers. *Open participation* could overcome problems associated with editorial selection of reviewers (e.g. biases, closed-networks, elitism). However, in opening up participation to the wider community, it might actually increase engagement by those with conflicts of interest. Where anonymity is possible, this may be particularly problematic. Moreover, lack of anonymity for reviewers in *open identities* review might subvert the process by discouraging reviewers from making strong criticisms, especially against higher-status colleagues.
- **Social and publication biases:** *Open reports* adds another layer of quality assurance, allowing the wider community to scrutinize reviews to examine decision-making processes. However, *open identities* removes anonymity conditions for reviewers (single-blind) or authors and reviewers (double-blind) which are traditionally in place to counteract social biases (although there is not strong-evidence that such anonymity has been effective).
- **Lack of incentives:** *Open reports* linked to *open identities* enable higher visibility for peer review activities, allowing review work to be cited in other publications and in career development activities linked to promotion and tenure. *Open participation* could in principle increase incentives to peer review by enabling reviewers to themselves select the works that they consider themselves qualified to judge; however in practice, experience to date suggests that reviewers are less likely to review under this condition.
- **Wastefulness:** *Open reports* make currently invisible but potentially useful scholarly information available for re-use, as well as providing young researchers a guide (to tone, length, the formulation of criticisms) to help them as they begin to do peer review themselves.

This synthesis allows us to draw the following conclusions: (1) the individual traits of OPR can be argued to address many of the problems with traditional peer review, but (2) differing traits addresses differing problems in differing ways, (3) no trait addresses all problems, and in fact (4) individual traits may actually exacerbate problems in some areas. Assessing this already complex landscape is made yet more problematic by the fact that (5) there is often little evidence to support or challenge many of these claims. There is hence a pressing need for more research to empirically evaluate the efficacy of differing traits in resolving these issues.

### Open Science as the unifying theme for the traits of OPR

The traits that we have identified to be part of definitions of OPR are disparate in their aims and implementation. Is there any common thread between them? I would argue yes: they each aim to bring peer review more into line with the emergent agenda of Open Science. To advance this argument, I’ll next briefly describe this movement and its underlying aims, and then relate each OPR trait to this agenda.

Open Science is the name given to a broad movement to reshape scholarly communication. As the English word “science” traditionally excludes the humanities and social sciences, the phenomenon is often referred to by more explicitly inclusive terms like “open scholarship” or “open research”. As “Open Science” is the more common term, I shall use it here, but should be read as referring to research from all academic disciplines.

**Figure 8.**
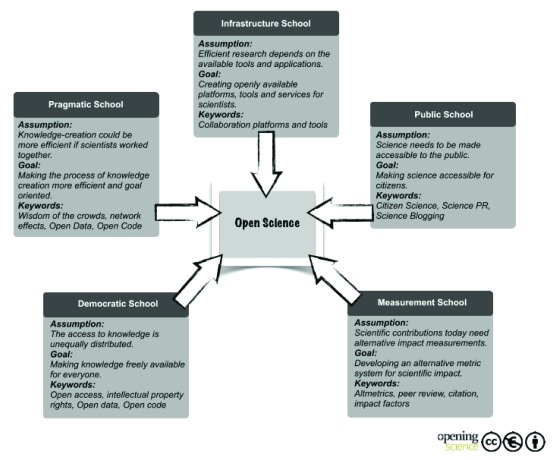
Five schools of thought in Open Science (CC BY-NC, Fecher & Friesike, 2013).

Open Science encompasses a variety of practices, usually including areas like open access to publications, open research data, open source software/tools, open workflows, citizen science, open educational resources, and alternative methods for research evaluation including open peer review ( Pontika *et al*., 2015). The aims and assumptions underlying the push to implement these various practices have been analysed by Fecher & Friesike (2013), whose analysis of the literature found five broad concerns, or “schools of thought” ( Figure 8). These are:

- **Democratic school**: Believing that there is an unequal distribution of access to knowledge, this area is concerned with making scholarly knowledge (including publications and data) available freely for all.
- **Pragmatic school:** Following the principle that the creation of knowledge is made more efficient through collaboration and strengthened through critique, this area seeks to harness network effects by connecting scholars and making scholarly methods transparent.
- **Infrastructure school:** This thread is motivated by the assumption that efficient research requires readily available platforms, tools and services for dissemination and collaboration.
- **Public school:** Based on the recognition that true societal impact requires societal engagement in research and readily understandable communication of scientific results, this area seeks to bring the public to collaborate in research through citizen science, and make scholarship more readily understandable through lay summaries, blogging and other less formal communicative methods.
- **Measurement school:** Motivated by the acknowledgement that traditional metrics for measuring scientific impact have proven problematic (by being too heavily focused on publications, often only at the journal-level, for instance), this strand seeks “alternative metrics” which can make use of the new possibilities of digitally networked tools to track and measure the impact of scholarship through formerly invisible activities.

The traits of OPR, in differing yet overlapping ways, each aim to bring greater transparency, accountability, inclusivity and/or efficiency to the restricted model of traditional peer review. The traits of OPR can be fit into Fecher & Friesike’s Open Science schema thus:

- **Democratic school**: *Open reports* further make scholarly products available to all.
- **Pragmatic school:** *Open identities* foster increased accountability by linking scholars’ names to their judgements; *open reports* increases transparency by opening review reports to readers; *open interaction* fosters increased collaboration between authors, reviewers and editors in the process of evaluation and revision of scholarship; *open pre-review manuscripts* enable the earlier dissemination of results.
- **Infrastructure school:** *Open platforms* can make peer review more efficient by decoupling it from journals.
- **Public school:** *Open participation* and *final-version commenting* bring greater inclusivity to peer review by expanding the potential pool of reviewers, including to those outside traditional research actors.
- **Measurement school:** *Open identities*, *open reports* and *open platforms* (e.g., Publons) enable peer review activities to be more clearly monitored and taken into account in impact-measurement activities.

## Conclusion

We have seen that the definition of “open peer review” is contested ground. My aim here has been to provide some clarity as to what is being referred to when this term is used. This is especially important since interest in the term (measured via references in the literature) is growing rapidly. By analyzing 122 separate definitions from the literature I have identified seven different traits of OPR, which all aim to resolve differing problems with traditional peer review. Amongst the corpus of definitions there are 22 unique configurations of these traits, meaning 22 distinct definitions of OPR in the reviewed literature. Across all definitions, the core elements are open identities and open reports, with one or both elements present in over 95% of the definitions examined. Among the other elements, open participation is the next most common element, and should perhaps be considered a core trait in SSH. Further secondary elements are open interaction and pre-review manuscripts. Fringe elements include open final version commenting and open platforms.

Given that OPR is such a contested concept, in my view the only sensible way forward is to acknowledge the ambiguity of this term, accepting that it is used as an umbrella concept for a diverse array of peer review innovations. Although it could be argued that merely accepting the status quo in this way does not help resolve possible confusion regarding usage, I would argue that quantifying the ambiguity of usage and mapping the distinct traits enables future discussion to start from a firmer basis that (1) acknowledges that people often mean different things when they use this term, and (2) clarifies in advance exactly which OPR traits are under discussion.

By being clear about these distinct traits, it will enable us to treat the ambiguity of OPR as a feature and not a bug. The large number of possible configurations of options presents a tool-kit for differing communities to construct open peer review systems that reflect their own needs, preferences and goals. The finding that there seems to be a difference in interpretations between disciplines (for example, that open participation seems more central to conceptions of OPR in SSH than STEM) reinforces this view. Moreover, disambiguating these traits will enable more focused analysis of the extent to which these traits are actually effective in countering the problems they are claimed to address. This is particularly urgent because, as we have seen, there is often little evidence to support or refute many of these claims.

Based upon this analysis I offer the following definition:

**OPR definition:** Open peer review is an umbrella term for a number of overlapping ways that peer review models can be adapted in line with the aims of Open Science, including making reviewer and author identities open, publishing review reports and enabling greater participation in the peer review process. The full list of traits is:

- **Open identities:** Authors and reviewers are aware of each other’s identity
- **Open reports:** Review reports are published alongside the relevant article.
- **Open participation:** The wider community to able to contribute to the review process.
- **Open interaction:** Direct reciprocal discussion between author(s) and reviewers, and/or between reviewers, is allowed and encouraged.
- **Open pre-review manuscripts:** Manuscripts are made immediately available (e.g., via pre-print servers like arXiv) in advance of any formal peer review procedures.
- **Open final-version commenting:** Review or commenting on final “version of record” publications.
- **Open platforms (“decoupled review”):** Review is facilitated by a different organizational entity than the venue of publication.

## Data availability

The data referenced by this article are under copyright with the following copyright statement: Copyright: © 2017 Ross-Hellauer T

Dataset including full data files used for analysis in this review: http://doi.org/10.5281/zenodo.438024 ( Ross-Hellauer, 2017).

## Notes

^1^This quote was found on the P2P Foundation Wiki ( http://wiki.p2pfoundation.net/Open_Peer_Review, accessed 18 ^th^ July 2016). Its provenance is uncertain, even to Suber himself, who recently advised in personal correspondence (19 ^th^ August 2016): “I might have said it in an email (as noted). But I can’t confirm that, since all my emails from before 2009 are on an old computer in a different city. It sounds like something I could have said in 2007. If you want to use it and attribute it to me, please feel free to note my own uncertainty!”

## Supplementary material

**Supplementary file 1: PRISMA checklist.** The checklist was completed with the original copy of the manuscript.

**Supplementary file 2: PRISMA flowchart showing the number of records identified, included and excluded.**

## References

[ref-1] Armstrong JS: Barriers to Scientific Contributions: The Authors Formula. *Behav Brain Sci.* Cambridge University Press (CUP). 1982;5(02):197–199 10.1017/s0140525x00011201

[ref-2] Armstrong JS: Peer Review for Journals: Evidence on Quality Control Fairness, and Innovation. *Sci Eng Ethics.* Springer Nature. 1997;3(1):63–84. 10.1007/s11948-997-0017-3

[ref-3] Bardy AH: Bias in reporting clinical trials. *Br J Clin Pharmacol.* Wiley-Blackwell. 1998;46(2):147–50. 10.1046/j.1365-2125.1998.00759.x 9723823 PMC1873669

[ref-4] Bloom T: Referee Report For: What is open peer review? A systematic review [version 1; referees: 1 approved, 3 approved with reservations]. *F1000Res.* 2017;6:588 10.5256/f1000research.12273.r22301 28580134 PMC5437951

[ref-5] Boldt A: Extending ArXiv.Org to Achieve Open Peer Review and Publishing. *J Scholarly Publ.* University of Toronto Press Inc. (UTPress), 2011;42(2):238–42. 10.3138/jsp.42.2.238

[ref-6] Bornmann L Herich H Joos H: In Public Peer Review of Submitted Manuscripts How Do Reviewer Comments Differ from Comments Written by Interested Members of the Scientific Community? A Content Analysis of Comments Written for *Atmospheric Chemistry and Physics*. *Scientometrics.* Springer Nature. 2012;93(3):915–29. 10.1007/s11192-012-0731-8

[ref-7] Budden AE Tregenza T Aarsen LW: Double-blind review favours increased representation of female authors. *Trends Ecol Evol.* 2008;23(1):4–6. 10.1016/j.tree.2007.07.008 17963996

[ref-8] Campanario JM: Peer Review for Journals as It Stands Today-Part 1. *Sci Commun.* SAGE Publications. 1998;19(3):181–211. 10.1177/1075547098019003002

[ref-9] Casadevall A Fang FC: Specialized science. *Infect Immun.* 2014;82(4):1355–1360. 10.1128/IAI.01530-13 24421049 PMC3993417

[ref-10] Cassella M Calvi L: New Journal Models and Publishing Perspectives in the Evolving Digital Environment. *IFLA Journal.* SAGE Publications. 2010;36(1):7–15. 10.1177/0340035209359559

[ref-11] Chubin DE Hackett EJ: Peerless Science: Peer Review and US Science Policy.Suny Press,1990 Reference Source

[ref-13] Cronin B: Vernacular and Vehicular Language. *J Am Soc Inf Sci Technol.* Wiley-Blackwell. 2009;60(3):433 10.1002/asi.21010

[ref-14] Dall’Aglio P: Peer Review and Journal Models.ArXiv:Physics/0608307,2006 Reference Source

[ref-15] Daniel HD: Guardians of Science.Wiley-Blackwell,1993 10.1002/3527602208

[ref-16] Day C: Meet the Overlay Journal. *Phys Today.*AIP Publishing,2015 10.1063/pt.5.010330

[ref-17] Dickersin K Min YI Meinert CL: Factors influencing publication of research results. Follow-up of applications submitted to two institutional review boards. *JAMA.* American Medical Association (AMA). 1992;267(3):374–8. 10.1001/jama.1992.03480030052036 1727960

[ref-18] EMBO Journal: About | The EMBO Journal [WWW Document].2016; (accessed 8.24.16). Reference Source

[ref-19] Ernst E Kienbacher T: Chauvinism. *Nature.* Springer Nature. 1991;352(6336):560 10.1038/352560b0

[ref-20] Fanelli D: Do Pressures to Publish Increase Scientists' Bias? An Empirical Support from US States Data.Edited by Enrico Scalas. *PLoS One.* Public Library of Science (PLoS). 2010;5(4):e10271. 10.1371/journal.pone.0010271 20422014 PMC2858206

[ref-21] Fang FC Casadevall A: Retracted Science and the Retraction Index. *Infect Immun.* American Society for Microbiology. 2011;79(10):3855–59. 10.1128/IAI.05661-11 21825063 PMC3187237

[ref-22] Fang FC Steen RG Casadevall A: Misconduct Accounts for the Majority of Retracted Scientific Publications. *Proc Natl Acad Sci U S A.* Proceedings of the National Academy of Sciences. 2012;109(42):17028–33. 10.1073/pnas.1212247109 23027971 PMC3479492

[ref-23] Fecher B Friesike S: Open Science: One Term, Five Schools of Thought.In: Bartling, S. and Friesike (Eds.), *Opening Science.*New York, NY: Springer,2013;17–47. 10.2139/ssrn.2272036

[ref-24] Fisher M Friedman SB Strauss B: The Effects of Blinding on Acceptance of Research Papers by Peer Review. *JAMA.* American Medical Association (AMA). 1994;272(2):143–46. 10.1001/jama.1994.03520020069019 8015127

[ref-25] Fitzpatrick K: Planned Obsolescence.New York, NY: NYU Press,2011 Reference Source

[ref-26] Fitzpatrick K Santo A: Open Review, A Study of Contexts and Practices. Report.2012 Reference Source

[ref-27] Ford E: Defining and Characterizing Open Peer Review: A Review of the Literature. *J Scholarly Publ.*University of Toronto Press Inc. (UTPress),2013;44(4):311–26. 10.3138/jsp.44-4-001

[ref-28] Ford E: Open peer review at four STEM journals: an observational overview [version 2; referees: 2 approved, 2 approved with reservations]. *F1000Res.*F1000 Research Ltd.2015;4:6. 10.12688/f1000research.6005.2 25767695 PMC4350441

[ref-29] Frontiers: About Frontiers Academic Journals and Research Community.2016 Reference Source

[ref-30] Garcia JA Rodriguez-Sanchez R Fdez-Valdivia J: Authors and Reviewers Who Suffer from Confirmatory Bias. *Scientometrics.*Springer Nature.2016;109(2):1377–95. 10.1007/s11192-016-2079-y

[ref-31] Gillespie GW Chubin DE Kurzon GM: Experience with NIH Peer Review: Researchers Cynicism and Desire for Change. *Sci Technol Hum Val.* 1985;10(3):44–54. 10.1177/016224398501000306

[ref-32] Godlee F Gale CR Martyn CN: Effect on the quality of peer review of blinding reviewers and asking them to sign their reports: a randomized controlled trial. *JAMA.*American Medical Association (AMA).1998;280(3):237–40. 10.1001/jama.280.3.237 9676667

[ref-33] Hames I: The Changing Face of Peer Review. *Sci Ed.*Korean Council of Science Editors.2014;1(1):9–12. 10.6087/kcse.2014.1.9

[ref-34] Hanson B Lawrence R Meadows A: Early Adopters of ORCID Functionality Enabling Recognition of Peer Review: Two Brief Case Studies. *Learn Publ.*Wiley-Blackwell.2016;29(1):60–63. 10.1002/leap.1004

[ref-35] Harnad S: The Invisible Hand of Peer Review.Journal (On-line/Unpaginated). Exploit Interactive.2000 Reference Source

[ref-36] Herron DM: Is expert peer review obsolete? A model suggests that post-publication reader review may exceed the accuracy of traditional peer review. *Surg Endosc.*Springer Nature.2012;26(8):2275–80. 10.1007/s00464-012-2171-1 22350231

[ref-37] Ioannidis JP: Effect of the statistical significance of results on the time to completion and publication of randomized efficacy trials. *JAMA.*American Medical Association (AMA).1998;279(4):281–6. 10.1001/jama.279.4.281 9450711

[ref-38] Janowicz K Hitzler P: Open and Transparent: the Review Process of the Semantic Web Journal. *Learn Publ.*Wiley-Blackwell.2012;25(1):48–55. 10.1087/20120107

[ref-39] Jubb M: Peer Review: The Current Landscape and Future Trends. *Learn Publ.*Wiley-Blackwell.2016;29(1):13–21. 10.1002/leap.1008

[ref-40] Justice AC Cho MK Winker MA: Does masking author identity improve peer review quality? A randomized controlled trial. PEER Investigators. *JAMA.*American Medical Association (AMA).1998;280(3):240–2. 10.1001/jama.280.3.240 9676668

[ref-41] Kaplan S: Major Publisher Retracts 64 Scientific Papers in Fake Peer Review Outbreak.Washington Post,2015 Reference Source

[ref-42] Kerr S Tolliver J Petree D: Manuscript Characteristics Which Influence Acceptance for Management and Social Science Journals. *Acad Manage J.*The Academy of Management.1977;20(1):132–41. 10.2307/255467

[ref-43] Kravitz RL Franks P Feldman MD: Editorial peer reviewers' recommendations at a general medical journal: are they reliable and do editors care? *PLoS One.*Public Library of Science (PLoS).2010;5(4):e10072. 10.1371/journal.pone.0010072 20386704 PMC2851650

[ref-44] Kriegeskorte N: Open evaluation: a vision for entirely transparent post-publication peer review and rating for science. *Front Comput Neurosci.*Frontiers Media SA.2012;6:79. 10.3389/fncom.2012.00079 23087639 PMC3473231

[ref-45] Leek JT Taub MA Pineda FJ: Cooperation between referees and authors increases peer review accuracy. *PLoS One.*Public Library of Science (PLoS).2011;6(11):e26895. 10.1371/journal.pone.0026895 22096506 PMC3212530

[ref-46] Link AM: US and non-US submissions: an analysis of reviewer bias. *JAMA.* 1998;280(3):246–7. 10.1001/jama.280.3.246 9676670

[ref-47] Lloyd ME: Gender factors in reviewer recommendations for manuscript publication. *J Appl Behav Anal.*Society for the Experimental Analysis of Behavior.1990;23(4):539–43. 16795738 10.1901/jaba.1990.23-539 PMC1286270

[ref-48] Mahoney MJ: Publication Prejudices: An Experimental Study of Confirmatory Bias in the Peer Review System. *Cognit Ther Res.* 1977;1(2): Springer Nature:161–75. 10.1007/BF01173636

[ref-49] McNutt RA Evans AT Fletcher RH: The effects of blinding on the quality of peer review. A randomized trial. *JAMA.*American Medical Association (AMA).1990;263(10):1371–6. 10.1001/jama.1990.03440100079012 2304216

[ref-50] Monsen ER Van Horn L: Research: Successful Approaches. American Dietetic Association;2007 Reference Source

[ref-51] Moore S Neylon C Eve MP: Excellence R Us: University Research and the Fetishisation of Excellence. *Palgrave Commun.*Springer Nature.2017;3: 16105. 10.1057/palcomms.2016.105

[ref-52] Mulligan A Hall L Raphael E: Peer Review in a Changing World: An International Study Measuring the Attitudes of Researchers. *J Am Soc Inf Sci Technol.*Wiley-Blackwell.2013;64(1):132–61. 10.1002/asi.22798

[ref-53] Nicholson J Alperin JP: A Brief Survey on Peer Review in Scholarly Communication. The Winnower,2016 Reference Source

[ref-54] Nickerson RC Varshney U Muntermann J: A Method for Taxonomy Development and Its Application in Information Systems. *Eur J Inf Syst.*Springer Nature.2013;22(3):336–59. 10.1057/ejis.2012.26

[ref-55] Nobarany S Booth KS: Use of Politeness Strategies in Signed Open Peer Review. *J Assoc Inf Sci Technol.*Wiley-Blackwell.2015;66(5):1048–64. 10.1002/asi.23229

[ref-56] OpenAIRE: OpenAIRE’s Experiments in Open Peer Review / Report. *Zenodo.* 2016 10.5281/zenodo.154647

[ref-57] Perakakis P Taylor M Mazza M: Natural Selection of Academic Papers. *Scientometrics.*Springer Nature.2010;85(2):553–59. 10.1007/s11192-010-0253-1

[ref-58] Peters DP Ceci SJ: Peer-Review Practices of Psychological Journals: The Fate of Published Articles Submitted Again. *Behav Brain Sci.*Cambridge University Press (CUP).1982;5(02):187–195. 10.1017/S0140525X00011183

[ref-59] Pontika N Knoth P Cancellieri M: Fostering Open Science to Research Using a Taxonomy and an ELearning Portal. In *Proceedings of the 15th International Conference on Knowledge Technologies and Data-Driven Business - i-KNOW 15* Association for Computing Machinery (ACM).2015 10.1145/2809563.2809571

[ref-60] Pöschl U: Multi-stage open peer review: scientific evaluation integrating the strengths of traditional peer review with the virtues of transparency and self-regulation. *Front Comput Neurosci.*Frontiers Media SA.2012;6:33. 10.3389/fncom.2012.00033 22783183 PMC3389610

[ref-61] PubPeer: Science Self-Corrects – Instantly. PubPeer: The Online Journal Club.2014 Reference Source

[ref-62] Research Information Network: Activities, Costs and Funding Flows in the Scholarly Communications System in the UK: Report Commissioned by the Research Information Network (RIN).2008 Reference Source

[ref-63] Ross JS Gross CP Desai MM: Effect of blinded peer review on abstract acceptance. *JAMA.*American Medical Association (AMA).2006;295(14):1675–80. 10.1001/jama.295.14.1675 16609089

[ref-64] Ross-Hellauer T: Review of Definitions of Open Peer Review in the Scholarly Literature 2016.2017 Data Source

[ref-65] Sandewall E: Maintaining Live Discussion in Two-Stage Open Peer Review. *Front Comput Neurosci.*Frontiers Media SA.2012;6:9. 10.3389/fncom.2012.00009 22363282 PMC3282940

[ref-66] Schekman R Watt F Weigel D: The *eLife* approach to peer review. *eLife.*eLife Sciences Organisation Ltd.2013;2:e00799. 10.7554/eLife.00799 23638304 PMC3639505

[ref-67] Schroter S Black N Evans S: Effects of Training on Quality of Peer Review: Randomised Controlled Trial. *BMJ.*BMJ.2004;328(7441):673–70. 10.1136/bmj.38023.700775.AE 14996698 PMC381220

[ref-68] Smith R: Peer review: a flawed process at the heart of science and journals. *J R Soc Med.*SAGE Publications.2006;99(4):178–82. 16574968 10.1258/jrsm.99.4.178 PMC1420798

[ref-69] Spier R: The History of the Peer-Review Process. *Trends Biotechnol.*Elsevier BV.2002;20(8):357–58. 10.1016/S0167-7799(02)01985-6 12127284

[ref-70] Steen RG Casadevall A Fang FC: Why has the number of scientific retractions increased?Edited by Gemma Elizabeth Derrick. *PLoS One.*Public Library of Science (PLoS).2013;8(7):e68397. 10.1371/journal.pone.0068397 23861902 PMC3704583

[ref-71] Suber P: Open Access.Cambridge, MA: MIT Press,2012 Reference Source

[ref-80] Tennant JP Dugan JM Graziotin D: A multi-disciplinary perspective on emergent and future innovations in peer review [version 1; referees: 2 approved with reservations]. *F1000Res.* 2017;6:1151 10.12688/f1000research.12037.1 PMC5686505 29188015

[ref-72] Travis GD Collins HM: New Light on Old Boys: Cognitive and Institutional Particularism in the Peer Review System. *Sci Technol Hum Val.* 1991;16(3). 10.1177/016224399101600303

[ref-73] Tregenza T: Gender Bias in the Refereeing Process? *Trends Ecol.* 2002;17(8):349–350. 10.1016/S0169-5347(02)02545-4

[ref-74] van Rooyen S Delamothe T Evans SJ: Effect on peer review of telling reviewers that their signed reviews might be posted on the web: randomised controlled trial. *BMJ.*BMJ.2010;341:c5729. 10.1136/bmj.c5729 21081600 PMC2982798

[ref-75] van Rooyen S Godlee F Evans S: Effect of open peer review on quality of reviews and on reviewers' recommendations: a randomised trial. *BMJ.*BMJ.1999;318(7175):23–27. 10.1136/bmj.318.7175.23 9872878 PMC27670

[ref-76] Walker R Rocha da Silva P: Emerging trends in peer review-a survey. *Front Neurosci.*Frontiers Media SA.2015;9:169. 10.3389/fnins.2015.00169 26074753 PMC4444765

[ref-77] Ware M: Peer Review: Benefits, Perceptions and Alternatives. Publishing Research Consortium 4,2008 Reference Source

[ref-78] Ware M: Peer Review: Recent Experience and Future Directions. *New Review of Information Networking.*Informa UK Limited.2011;16(1):23–53. 10.1080/13614576.2011.566812

[ref-79] Ware M: Peer Review Survey 2015. Publishing Research Consortium.2016 Reference Source

