# Supplementary material for: What is open peer review? A systematic review
Source: F1000Res. 2017 Aug 31;6:588. Originally published 2017 Apr 27. [Version 2] doi: 10.12688/f1000research.11369.2 (PMC5437951; doi:10.12688/f1000research.11369.2)
Supplement: Supplementary file 2 [file f1000research-6-13517-s0001.tgz › 913a1ac5-bbdf-4a4b-8b03-8da8b47927cb.doc]

**PRISMA flow diagram for systematic review of definitions of “open peer review”**

**Screening**

**Included**

**Eligibility**

**Identification**

Records identified through database searching Web of Science (searched for: TOPIC: ("open review" OR "open peer review"), 12-7-2016)
(n = 137)

Additional records identified through other sources (mining bibliographies, relevant literature reviews, searching Google Books and Google)
(n = 42)

Records after duplicates removed
(n = 174)

Records screened
(n = 174)

Records excluded
(n = 7)

Not relevant, not in English language (n = 7)

Full-text articles assessed for eligibility
(n = 167)

Full-text articles excluded
(n = 45)

- Not relevant, peer review of objects outside of stated scope (e.g., standards, physiological workflows, etc.) (n = 12)
- Not relevant, “open review” refers to literature review/metastudy article, not peer review (n = 12)
- Not relevant, article has been under open peer review, but is not about open peer review (n = 21)

Studies included in qualitative synthesis
(n = 122)

*Adapted from:*  Moher D, Liberati A, Tetzlaff J, Altman DG, The PRISMA Group (2009). *P*referred *R*eporting *I*tems for *S*ystematic Reviews and *M*eta-*A*nalyses: The PRISMA Statement. PLoS Med 6(7): e1000097. doi:10.1371/journal.pmed1000097
