# Supplementary material for: What is open peer review? A systematic review
Source: F1000Res. 2017 Aug 31;6:588. Originally published 2017 Apr 27. [Version 2] doi: 10.12688/f1000research.11369.2 (PMC5437951; doi:10.12688/f1000research.11369.2)
Supplement: Peer review history

# Peer review: Referee response for version 2

**DOI:** 10.5256/f1000research.13517.r25574

**Published:** 2017-10-10

**Authors:** Bahar Mehmani

**Affiliations:** 1 Global Publishing Development department, Elsevier, RELX Group, Amsterdam, Netherlands

I read the new version and confirm all my comments to the original version are now addressed properly in the article.

 I have no further comments and approve the current version of the article.

I have read this submission. I believe that I have an appropriate level of expertise to confirm that it is of an acceptable scientific standard.

# Peer review: Referee response for version 2

**DOI:** 10.5256/f1000research.13517.r25576

**Published:** 2017-10-04

**Authors:** Theodora Bloom

**Affiliations:** 1 The BMJ, London, UK

I commend the author for a thorough revision of the manuscript in the light of reviewers' comments. 

 I had three significant concerns previously. The first was about methodology, and the author has addressed this with more details and description of the method. The second concerned omitting mention of 'real time peer review in the open' as an option, and including 'open platforms'. Realtime open review is now mentioned. Open platforms are retained, but it is mentioned that they are an unusual (or 'fringe') case of the meaning of open peer review. The final major concern was about conflating editorial selection with peer review, and the author has now nicely clarified this distinction.  

 My more minor concerns have also all been addressed.

I have read this submission. I believe that I have an appropriate level of expertise to confirm that it is of an acceptable scientific standard.

# Peer review: Referee response for version 2

**DOI:** 10.5256/f1000research.13517.r25575

**Published:** 2017-09-26

**Authors:** Emily Ford

**Affiliations:** 1 Urban & Public Affairs Librarian, Portland State University, Portland, OR, USA

I commend the author on the edits and revisions to this article. They have strengthened the work. The expansion of the methods section, as well as the new figure and additional language discussing differences between STEM and SSH disciplines enhances this article and situates it for broader, cross-disciplinary discussion and impact. Further, these revisions have allowed the author to articulate areas for further study. 

 These revisions have addressed the majority of my previous comments. Those comments that were not address by the revisions pertaining to disciplinary economic disparities introduced by article processing charges as well as further delving into differences between STEM and SSH disciplines, are out of scope for this article. Related to the article's discussion of the "wastefulness" of traditional peer review models, my hope is that this thread, which introduces and discusses areas for future research along this vein, is captured in the scholarly conversation and written record.

I have read this submission. I believe that I have an appropriate level of expertise to confirm that it is of an acceptable scientific standard.

# Peer review: Referee response for version 1

**DOI:** 10.5256/f1000research.12273.r22576

**Published:** 2017-05-22

**Authors:** Emily Ford

**Affiliations:** 1 Urban & Public Affairs Librarian, Portland State University, Portland, OR, USA

Introduction

- The definition of open science needs to be clearly stated in the Introduction in order to strengthen the frame of the whole paper. Is the definition you are using of open science fully accepted and not contested? If so, then great, but if not, then it becomes murkier and you might want to spend time unpacking the tension there. Also in the last sentence of the Intro, what is that ethos of open science?

Introduction: Background

- Would it be useful to unpack some counter arguments on the reasons peer review in its current state of blinded does not work? For example, in the delay and expense portion, how does flipping the model to use APCs change the cost at all? And what happens to unfunded research when the model is flipped? Does that create a disparity that only well funded research is readily available? This might create yet another stratification of scholarly publishing and science communication, which one would assume open science is trying to diminish. I realize that you do some of this in the discussion section, but I find there is a gap in the discussion of the economic argument.

Introduction: Contested Meaning

- I appreciate your thoughtful criticisms of past works that have been unable to do what you are doing in this article. Being the author of one of them, however, I would like to make some points.
- Would like to point out that while I understand the lack of a definition in my authored article (Defining & Characterizing Open Peer Review - 2013) is problematic, it was never my intent to fully describe it, but I had to use a scope for my systematic review, and that scope was identity disclosure. Please note that in my concluding remarks on that paper that I recommended a definition be more tightly defined, and it never claimed to define it wholesale.
- Your research does a good job picking up the task that other papers were unable to accomplish.

Methodology

- I would like to hear more in your methodology section about the searching for and selection of social sciences and humanities literature, as I think there might be some gaps in your data set based on this approach. You provide your search terms for Web of Science, but not the other databases and search engines. Including this would strengthen your methodology section. To me, the treatment of social sciences and humanities in this study is one of its weaknesses.
- Please outline the limitations of your research method.
- The methods section should be strengthened for better understanding of social sciences and humanities approaches, as well as limitations, for the paper to be more scientifically sound.

Results

- I am not a statistician, nor am I a quantitative researcher, so I cannot provide a robust review of your results when it comes to these facets.
- Figures and tables are helpful to translate findings and ideas presented.

Discussion:

- This section is well organized and easy to understand. I appreciate the presentation of criticisms of OPR in this section.
- Open participation and open interaction sections would be greatly enhanced and do a great service to your consideration of the social sciences and humanities disciplines if you engaged with Fitzpatrick’s work presented in the Mellon White paper as well the Logos article. Additionally, there is an article not included in your data set (was it out of scope?), in Social Epistemology, that may help. It would be good to engage more deeply with the question: Is OPR changing the role and purpose of peer review itself? There seems to be evidence of this by the mentorship offered at eLife, the encouragement of reviewers to engage with one another at Frontiers, and generally by collaborative approaches to review that OPR enables. In my view these approaches make peer review more robust, including more than just vetting, fact checking, and some substantive critical feedback. To this end, you will need to more clearly define in your introduction and throughout the paper the assumed purpose of peer review, which you offer us in the open final-versioning commenting portion of the discussion section.
- Open platforms section: While I agree that today platforms are an enormous part of our work in communicating science and engaging with our colleagues across the globe. That being said, I would like to point out that the process of peer review could be completely decoupled from a platform. The reason I mention this is that for some individuals and perhaps some disciplines, it might be difficult to get one’s head around the distinction between a peer review process and its technological implementation. To me they are distinct, and it is merely digital technology that assists us in allowing OPR to unfold. It behooves shy away from techno determinism when it comes to the possibilities presented by OPR.

Conclusion

- I think you have a solid finding, but I would like to point out one more quibble. “Open science” is not a term embraced in the social sciences and humanities. Again, since you are couching your definition under the ethos of open science you will need to better describe open science, and make a bridge for social science and humanities disciplines. If an overarching definition of OPR is to be fully accepted by all disciplines, it needs to be inclusive of all of them. This is wherein the tension lies, where the community-based aspect of OPR in the social sciences and humanities (digital humanities?) are much more pronounced in the meaning making of the process. How can you better acknowledge the disciplinary tensions in the paper? Or would you like to scope your findings differently?

Final thoughts

- This paper is well written and organized logically, which make it quite readable and easy to follow. 
- The main weakness of your paper is the lack of nuance addressed between STEM and social sciences and humanities disciplines. Engaging in the the tension between the approaches to and understanding of peer review and OPR in different disciplines will greatly strengthen your paper.
- Presenting better the limitations of your method and clarifying your method as noted above will help scope the paper to be more scientifically sound.
- Finally, clearly define and scope Open Science so that your proposed definition is more understandable. This will greatly strengthen not only the paper, but the definition itself.

I have read this submission. I believe that I have an appropriate level of expertise to confirm that it is of an acceptable scientific standard, however I have significant reservations, as outlined above.

# Response

**Published:** 2017-08-31

**Authors:** Tony Ross-Hellauer

**Affiliations:** OpenAIRE / Uni. Goettingen, Germany

***Emily Ford: **“Introduction: The definition of open science needs to be clearly stated in the Introduction in order to strengthen the frame of the whole paper. Is the definition you are using of open science fully accepted and not contested? If so, then great, but if not, then it becomes murkier and you might want to spend time unpacking the tension there. Also in the last sentence of the Intro, what is that ethos of open science?”*

**Tony Ross-Hellauer: **I'd like to thank the reviewer for their very thoughtful and helpful comments. The inclusion of more consideration of the SSH perspective, especially, definitely strengthens the paper.

***EF: **“Introduction: Background - Would it be useful to unpack some counter arguments on the reasons peer review in its current state of blinded does not work? For example, in the delay and expense portion, how does flipping the model to use APCs change the cost at all? And what happens to unfunded research when the model is flipped? Does that create a disparity that only well funded research is readily available? This might create yet another stratification of scholarly publishing and science communication, which one would assume open science is trying to diminish. I realize that you do some of this in the discussion section, but I find there is a gap in the discussion of the economic argument.”*

**TRH: **Open Participation relies to an extent on OA (have added a sentence on this), but I’m afraid I don't see a further connection here. Although OPR is of course related to OA journals (in that they have tended to be more likely to experiment with OPR), surely if the same system of (traditional, blinded) peer review is in use, the basic costs (for review) will be the same? I agree that a fully-APC based OA model of publishing has the potential to exclude less well-resourced institutions (especially outside the developed West), but do not follow how this wider argument is connected to OPR. In any case, I believe these considerations fall out of scope of this review (although it would be interesting to follow them up elsewhere).

*EF: "Introduction: Contested Meaning - I appreciate your thoughtful criticisms of past works that have been unable to do what you are doing in this article. Being the author of one of them, however, I would like to make some points. - would like to point out that while I understand the lack of a definition in my authored article (Defining & Characterizing Open Peer Review - 2013) is problematic, it was never my intent to fully describe it, but I had to use a scope for my systematic review, and that scope was identity disclosure. Please note that in my concluding remarks on that paper that I recommended a definition be more tightly defined, and it never claimed to define it wholesale. - Your research does a good job picking up the task that other papers were unable to accomplish."*

TRH: Thanks for clarifying this.

*EF “Methodology - I would like to hear more in your methodology section about the searching for and selection of social sciences and humanities literature, as I think there might be some gaps in your data set based on this approach. You provide your search terms for Web of Science, but not the other databases and search engines. Including this would strengthen your methodology section. To me, the treatment of social sciences and humanities in this study is one of its weaknesses. Please outline the limitations of your research method. The methods section should be strengthened for better understanding of social sciences and humanities approaches, as well as limitations, for the paper to be more scientifically sound.”*

TRH: I have expanded the methodology section to better specify search terms and databases used, and included a statement regarding limitations of the search strategy.

*EF: “Results - I am not a statistician, nor am I a quantitative researcher, so I cannot provide a robust review of your results when it comes to these facets. Figures and tables are helpful to translate findings and ideas presented.”*

TRH: No response required.

*EF: “Open participation and open interaction sections would be greatly enhanced and do a great service to your consideration of the social sciences and humanities disciplines if you engaged with Fitzpatrick’s work presented in the Mellon White paper as well the Logos article. Additionally, there is an article not included in your data set (was it out of scope?), in Social Epistemology (http://dx.doi.org/10.1080/02691728.2010.498929), that may help. It would be good to engage more deeply with the question: Is OPR changing the role and purpose of peer review itself? There seems to be evidence of this by the mentorship offered at eLife, the encouragement of reviewers to engage with one another at Frontiers, and generally by collaborative approaches to review that OPR enables. In my view these approaches make peer review more robust, including more than just vetting, fact checking, and some substantive critical feedback. To this end, you will need to more clearly define in your introduction and throughout the paper the assumed purpose of peer review, which you offer us in the open final-versioning commenting portion of the discussion section.”*

TRH: I have added consideration of disciplinary differences in the results and discussion sections. In particular, I have added a new figure to show the breakdown of traits by discipline, and added more consideration of the philosophical reasons to consider open participation and interaction. The question of whether OPR is changing the role of peer review per se is an excellent one, but I feel it is out of scope for this paper (which is already long enough!). The article by Fitzpatrick in Social Epistemology was deemed out of scope as it does not mention OPR (save for one mention in a block of quoted text) - the ideas underlying "peer-to-peer review" are no doubt related to the idea of OPR, but the scope here is only those papers which discuss OPR and give an explicit or implicit definition.

*EF: “Open platforms section: While I agree that today platforms are an enormous part of our work in communicating science and engaging with our colleagues across the globe. That being said, I would like to point out that the process of peer review could be completely decoupled from a platform. The reason I mention this is that for some individuals and perhaps some disciplines, it might be difficult to get one’s head around the distinction between a peer review process and its technological implementation. To me they are distinct, and it is merely digital technology that assists us in allowing OPR to unfold. It behooves shy away from techno determinism when it comes to the possibilities presented by OPR.”*

TRH: This is an important point - I have added a sentence to the section on final version commenting "In this sense, peer review can be decoupled not only from the journal, but also from any particular platform."

*EF: “Conclusion - I think you have a solid finding, but I would like to point out one more quibble. “Open science” is not a term embraced in the social sciences and humanities. Again, since you are couching your definition under the ethos of open science you will need to better describe open science, and make a bridge for social science and humanities disciplines. If an overarching definition of OPR is to be fully accepted by all disciplines, it needs to be inclusive of all of them. This is wherein the tension lies, where the community-based aspect of OPR in the social sciences and humanities (digital humanities?) are much more pronounced in the meaning making of the process. How can you better acknowledge the disciplinary tensions in the paper? Or would you like to scope your findings differently?“*

TRH: I have added an explicit reference to Open Science, specifically noting that I use the term to include all academic disciplines. I've also added reference to the disciplinary differences found, and included a concluding note that this area needs further research.

*EF: “Final thoughts - This paper is well written and organized logically, which make it quite readable and easy to follow. The main weakness of your paper is the lack of nuance addressed between STEM and social sciences and humanities disciplines. Engaging in the the tension between the approaches to and understanding of peer review and OPR in different disciplines will greatly strengthen your paper. Presenting better the limitations of your method and clarifying your method as noted above will help scope the paper to be more scientifically sound. Finally, clearly define and scope Open Science so that your proposed definition is more understandable. This will greatly strengthen not only the paper, but the definition itself.”*

TRH: Restatement of the above points - see answers above.

# Peer review: Referee response for version 1

**DOI:** 10.5256/f1000research.12273.r22575

**Published:** 2017-05-15

**Authors:** Bahar Mehmani

**Affiliations:** 1 Global Publishing Development department, Elsevier, RELX Group, Amsterdam, Netherlands

Tony provides an overview of different definitions of Open Peer Review, acknowledging the ambiguity of the term “open peer review” and the probable impact of such ambiguity on evaluation of the efficiency of open peer review. The author has created then seven OPR traits based on WoS data driven taxonomy. In conclusion though he suggests accepting the existing ambiguity is the only way: “given this is such a contested concept, in my view the only sensible way forward is to acknowledge the ambiguity of this term, accepting that it is used as an umbrella concept for a diverse array of peer review innovations”. This doesn’t seem to be solving the issue he raises at in beginning. On the contrary considering all peer review innovations as forms of OPR might worsen the current situation. Initiatives like “registered reports”, “shortening review deadlines” or “reviewer recognition”, which are meant to address result-biased peer review, incentivizing reviewers, and boosting review speed, respectively, are certainly peer review innovations but cannot be directly considered as part of the umbrella term of open peer review. Hence the article deserves a stronger conclusion, including perhaps a suggested guideline of clarifying what type of “open peer review” beforehand of any evaluation/discussion using authors seven classifications.

 Author can surely strengthen the conclusion by highlighting the lack of evidence in efficiency claims about some of the 7 traits mentioned in the article.

 I suggest a double checking the term “Post-publication peer review” in WoS search results. Author reports he has used “Open Final version commenting” for this process in making taxonomy reporting only 6 results. However, Post-publication peer review seem to produce a higher number of documents when I searched the term in Scopus results in 55 documents (11 for 2015 and 19 for 2016). This might impact the result reported in Fig. 6 reporting unique configurations of OPR. Although the author reports 22 different definitions there, the figures shows 23 of them.

 It is also worth mentioning most of OPR initiatives mentioned in the article are not directly addressing all of shortcomings of the current peer review process. Mainly because one should distinguish between editorial process and peer review process. Issues such as delay and expense are universal to single/double/open peer review as they are part of the editorial process.

I have read this submission. I believe that I have an appropriate level of expertise to confirm that it is of an acceptable scientific standard, however I have significant reservations, as outlined above.

# Response

**Published:** 2017-08-31

**Authors:** Tony Ross-Hellauer

**Affiliations:** OpenAIRE / Uni. Goettingen, Germany

***Bahar Mehmani:*** *„Tony provides an overview of different definitions of Open Peer Review, acknowledging the ambiguity of the term “open peer review” and the probable impact of such ambiguity on evaluation of the efficiency of open peer review. The author has created then seven OPR traits based on WoS data driven taxonomy. In conclusion though he suggests accepting the existing ambiguity is the only way: “given this is such a contested concept, in my view the only sensible way forward is to acknowledge the ambiguity of this term, accepting that it is used as an umbrella concept for a diverse array of peer review innovations”. This doesn’t seem to be solving the issue he raises at in beginning. On the contrary considering all peer review innovations as forms of OPR might worsen the current situation. Initiatives like “registered reports”, “shortening review deadlines” or “reviewer recognition”, which are meant to address result-biased peer review, incentivizing reviewers, and boosting review speed, respectively, are certainly peer review innovations but cannot be directly considered as part of the umbrella term of open peer review. Hence the article deserves a stronger conclusion, including perhaps a suggested guideline of clarifying what type of “open peer review” beforehand of any evaluation/discussion using authors seven classifications.”*

**Tony Ross-Hellauer:** I thank the reviewer for their time and care in preparing this helpful review report, which has helped to improve the second version. I understand the concern about merely accepting ambiguity to be no way to overcome confusion. I share this view and have now added/altered text to make my intentions clearer - "Given that OPR is such a contested concept, in my view the only sensible way forward is to acknowledge the ambiguity of this term, accepting that it is used as an umbrella concept for a diverse array of peer review innovations. Although it could be argued that merely accepting the status quo in this way does not help resolve possible confusion regarding usage, I would argue that quantifying the ambiguity of usage and mapping the distinct traits enables future discussion to start from a firmer basis that (1) acknowledges that people often mean different things when they use this term, and (2) clarifies in advance exactly which OPR traits are under discussion."

***BM:*** *“Author can surely strengthen the conclusion by highlighting the lack of evidence in efficiency claims about some of the 7 traits mentioned in the article.”*

**TRH:** I have included this point in the expanded conclusion.

*BM: “I suggest a double checking the term “Post-publication peer review” in WoS search results. Author reports he has used “Open Final version commenting” for this process in making taxonomy reporting only 6 results. However, Post-publication peer review seem to produce a higher number of documents when I searched the term in Scopus results in 55 documents (11 for 2015 and 19 for 2016). This might impact the result reported in Fig. 6 reporting unique configurations of OPR.”*

TRH: Although there is obvious overlap between the terms "open peer review" and "post-publication peer review", and so the literature on PPPR is useful to understand these phenomena, this study is only an attempt to categorize uses of the former term - hence, articles on PPPR which did not mention OPR  were out of scope.

*BM: “Although the author reports 22 different definitions there, the figures shows 23 of them.”*

TRH: The original figure was wrong and has been corrected. The final row of the original figure was a duplicate of the 12th entry (n=1, open reports, participation, manuscripts). I thank the reviewer very much for their attention to detail in spotting this silly error! Hence, it is correct that there are 22 different definitions.

*BM: “It is also worth mentioning most of OPR initiatives mentioned in the article are not directly addressing all of shortcomings of the current peer review process. Mainly because one should distinguish between editorial process and peer review process. Issues such as delay and expense are universal to single/double/open peer review as they are part of the editorial process.”*

TRH: I address this point with added text in the background and conclusion sections.

# Peer review: Referee response for version 1

**DOI:** 10.5256/f1000research.12273.r22301

**Published:** 2017-05-11

**Authors:** Theodora Bloom

**Affiliations:** 1 The BMJ, London, UK

This is an interesting paper addressing a question that is important to journal editors and publishers as well as the wider ‘open science’ community, namely what is meant by open peer review. I have three significant concerns that need to be addressed, followed by more minor annotations and comments.

 Major issues, in the order they arise in the article

  

1. Would this meet most established criteria for a systematic review? Although the author completes a PRISMA checklist, he also notes firstly that he searched Web of Science, then that he added a bunch of other sources, and finally, “This set of articles was further enriched with 42 definitions from sources found through searching for the same terms in other academic databases (e.g., Google Scholar, JSTOR, disciplinary databases), Google (for blog articles) and Google Books (for books), as well as following citations in relevant bibliographies and literature reviews." This suggests that it is not really systematic (although the author is to be applauded for providing the data he worked from). There is a lack of clarity about the universe of literature that is being assessed, and of details about how it was assessed. In my view, the article is still a worthwhile undertaking, despite being non-systematic, but the title ought to reflect this.
2. In my view the author does not pay enough attention to one important variant of open review, namely real-time review in the open, in which either invited reviewers or ‘the crowd’ comment on an article, with comments being posted as they are ready, rather than at the end of a formal process of peer review and decision-making. Conversely, the inclusion of ‘open platforms’ seems very confusing to me, as they do not have many of the criteria of openness that define the other flavours of open review that the author describes, but instead are about decoupling peer review from publication. Indeed Figure 6 makes plain that as a trait open platforms only occur twice in the 122 definitions considered. I would bet that the ‘real-time’ variant occurs far more often, and ought to be included as one of the key traits within the umbrella definition.
3. In a paper that aims to define open peer review, it is unfortunate that the author doesn’t spend longer considering alternative definitions of peer review. Throughout the article he appears to conflate editorial selection (whether a journal accepts a manuscript) with technical review (whether the work is sound and properly reported). Thus when he talks about the “problems with peer review” he is sometimes talking about reviewers not spotting technical problems, sometimes about editors rejecting articles that don’t suit their taste, and sometimes about authors going through cycles of editorial rejection to achieve a high impact publication. Conflating these various things does not provide a sound foundation on which to build a definition of open peer review. This conflation is made worse when, for example, it is implied that the only reason for retraction is error. Thus most of the first three paragraphs of the ‘background’ section, and much of what follows about biases, incentives and wastefulness are muddled, and the references and evidence do not all support the broad claims made about ‘peer review’ (itself an ‘umbrella term’).

 

 More minor concerns, with page numbers (PDF version) of the corresponding text

- Abstract and later: The author needs to decide throughout the article whether he is singular (as he appears to be) or uses the royal ‘we’.
- p7 – it is not helpful to list the seven types of openness without definitions. Even if the lengthy discussion of them follows later, I was desperate for some brief definition, in particular for the last two, and was labouring under a misapprehension about the meaning of the fourth, until much later in the article when I discovered what was meant by ‘open pre-review manuscripts’.  
- p8 – proponents of open identity review in medicine would also point out that it makes conflicts of interest much more apparent and subject to scrutiny.
- p8 – some journals use open reports without open identities – i.e. posting reports with published articles but without identifying the reviewer (e.g. http://embor.embopress.org/about#Transparent_Process). The author writes as if open reports must always have open identities.
- p10 – I think ‘open pre-review manuscripts’ is the wrong name for what the author is describing. At first I thought this meant the practice of posting the authors’ version of a manuscript alongside the peer review history (as is done by The BMJ, for example). But I think the author means ‘Open posting before formal review’ (which some call preprints). He might like to consider the suggestions by Neylon *et al. *about this issue of terminology (http://biorxiv.org/content/early/2016/12/09/092817)
- p10 – I wonder if the author has any evidence that PubPeer has been ‘a major influence’?
- p11 – I don’t think ‘open platforms’ is the right term either. (Although as noted above, I don’t really think this section belongs in the discussion at all, if it is to remain I would strongly recommend renaming it.) In publishing terms a platform is ‘where’ you publish articles, and the author is here discussing an aspect of how you get to the point of publication, and in particular peer review services (which as far as I can tell de facto meet only rather limited criteria of openness). I think what the author is describing is peer review options decoupled from journals (see Priem and Hemminger, Front Comput Neurosci 2012; 6: 19) and as noted I don’t understand why these have a place in a definition of open peer review.
- p11 – Conclusion. I don’t believe the author presents a unified definition of open peer review, for all the reasons discussed above, but he does present most of the traits that together come under the umbrella term.

I have read this submission. I believe that I have an appropriate level of expertise to confirm that it is of an acceptable scientific standard, however I have significant reservations, as outlined above.

# Response

**Published:** 2017-08-31

**Authors:** Tony Ross-Hellauer

**Affiliations:** OpenAIRE / Uni. Goettingen, Germany

***Theodora Bloom:*** *“This is an interesting paper addressing a question that is important to journal editors and publishers as well as the wider ‘open science’ community, namely what is meant by open peer review. I have three significant concerns that need to be addressed, followed by more minor annotations and comments.”*

**Tony Ross-Hellauer:** I'd like to thank the reviewer for their time and care in undertaking this review, which has helped strengthen the paper, especially with regards to the suggestions for strengthening the analysis of the problems with peer review.

***TB:*** *Would this meet most established criteria for a systematic review? Although the author completes a PRISMA checklist, he also notes firstly that he searched Web of Science, then that he added a bunch of other sources, and finally, “This set of articles was further enriched with 42 definitions from sources found through searching for the same terms in other academic databases (e.g., Google Scholar, JSTOR, disciplinary databases), Google (for blog articles) and Google Books (for books), as well as following citations in relevant bibliographies and literature reviews." This suggests that it is not really systematic (although the author is to be applauded for providing the data he worked from). There is a lack of clarity about the universe of literature that is being assessed, and of details about how it was assessed. In my view, the article is still a worthwhile undertaking, despite being non-systematic, but the title ought to reflect this.*

**TRH:** I have strengthened the description in the methods section to address this criticism. I do believe this article meets the criteria for PRISMA systematic reviews (the relevant sections from the PRISMA checklist being: "7. - Describe all information sources (e.g., databases with dates of coverage, contact with study authors to identify additional studies) in the search and date last searched"; and "8. Present full electronic search strategy for at least one database, including any limits used, such that it could be repeated." )

*TB: “In my view the author does not pay enough attention to one important variant of open review, namely real-time review in the open, in which either invited reviewers or ‘the crowd’ comment on an article, with comments being posted as they are ready, rather than at the end of a formal process of peer review and decision-making. Conversely, the inclusion of ‘open platforms’ seems very confusing to me, as they do not have many of the criteria of openness that define the other flavours of open review that the author describes, but instead are about decoupling peer review from publication. Indeed Figure 6 makes plain that as a trait open platforms only occur twice in the 122 definitions considered. I would bet that the ‘real-time’ variant occurs far more often, and ought to be included as one of the key traits within the umbrella definition.”*

TRH: I thank the reviewer for pointing out the gap in not mentioning what they term "real-time review in the open" - but I would respectfully disagree with the reviewer that this feature constitutes a core trait. In my view it depends upon other traits, including open reports, commenting, participation and especially open pre-review manuscripts. As this feature depends (in my view) most fully upon open pre-review manuscripts (since the manuscript would need to be online to begin the process), I have included mention of this option in the discussion section for that trait with the added text: "Finally, making manuscripts openly available in advance of review allows comments to be posted as they are received, either from invited reviewers or the wider community, and enabling readers to follow the process of peer-review in real-time."

*TB: “In a paper that aims to define open peer review, it is unfortunate that the author doesn’t spend longer considering alternative definitions of peer review. Throughout the article he appears to conflate editorial selection (whether a journal accepts a manuscript) with technical review (whether the work is sound and properly reported). Thus when he talks about the “problems with peer review” he is sometimes talking about reviewers not spotting technical problems, sometimes about editors rejecting articles that don’t suit their taste, and sometimes about authors going through cycles of editorial rejection to achieve a high impact publication. Conflating these various things does not provide a sound foundation on which to build a definition of open peer review. This conflation is made worse when, for example, it is implied that the only reason for retraction is error. Thus most of the first three paragraphs of the ‘background’ section, and much of what follows about biases, incentives and wastefulness are muddled, and the references and evidence do not all support the broad claims made about ‘peer review’ (itself an ‘umbrella term’).”*

TRH: Since the aim of this article was to clarify, I am very grateful to the reviewer for pointing out the ways in which this section actually confuses things. It was certainly not my intention to insinuate that OPR is a panacea for all problems with traditional peer review. I have added text to address the reviewer's comments: (a) The description of traditional peer review in the Background section has been revised to clarify the role of peer review in scholarly communication; (b) Two new sections have been added to the discussion which make clearer (1) the particular problems with traditional peer review that each OPR trait aims to address, and (2) how each trait can be related to the broader agenda of Open Science (a new figure is also added).

*TB: “Abstract and later: The author needs to decide throughout the article whether he is singular (as he appears to be) or uses the royal ‘we’.”*

TRH: Corrected

*TB: “p7 – it is not helpful to list the seven types of openness without definitions. Even if the lengthy discussion of them follows later, I was desperate for some brief definition, in particular for the last two, and was labouring under a misapprehension about the meaning of the fourth, until much later in the article when I discovered what was meant by ‘open pre-review manuscripts’. “*

TRH: Definitions added.

*TB: “p8 – proponents of open identity review in medicine would also point out that it makes conflicts of interest much more apparent and subject to scrutiny.”*

TRH: Added text to address this: "Finally, a reviewer for this paper advises that “proponents of open identity review in medicine would also point out that it makes conflicts of interest much more apparent and subject to scrutiny” (Bloom, 2017). "

*TB: “p8 – some journals use open reports without open identities – i.e. posting reports with published articles but without identifying the reviewer (e.g. http://embor.embopress.org/about#Transparent_Process). The author writes as if open reports must always have open identities.”*

TRH: Added sentence to clarify: "Often, although not in all cases (e.g., EMBO reports, http://embor.embopress.org), review names are published alongside the reports. "

*TB: “p10 – I think ‘open pre-review manuscripts’ is the wrong name for what the author is describing. At first I thought this meant the practice of posting the authors’ version of a manuscript alongside the peer review history (as is done by The BMJ, for example). But I think the author means ‘Open posting before formal review’ (which some call preprints). He might like to consider the suggestions by Neylon et al. about this issue of terminology (http://biorxiv.org/content/early/2016/12/09/092817)”*

TRH: I agree the terminology could be misconstrued, but am not sure the reviewer's suggestion is preferable (the phrase "posting" could be thought ambiguous) - to avoid ambiguity for future readers, I have added definitions on p7 where I introduce the terms.

*TB: “p10 – I wonder if the author has any evidence that PubPeer has been ‘a major influence’?”*

TRH: I’ve weakened the terminology to "An important platform in this regard has been major influence here has been the independent platform Pubpeer "

*TB: “p11 – I don’t think ‘open platforms’ is the right term either. (Although as noted above, I don’t really think this section belongs in the discussion at all, if it is to remain I would strongly recommend renaming it.) In publishing terms a platform is ‘where’ you publish articles, and the author is here discussing an aspect of how you get to the point of publication, and in particular peer review services (which as far as I can tell de facto meet only rather limited criteria of openness). I think what the author is describing is peer review options decoupled from journals (see Priem and Hemminger, Front Comput Neurosci 2012; 6: 19) and as noted I don’t understand why these have a place in a definition of open peer review.”*

TRH: I respectfully disagree with the reviewer here - the word "platform" seems to be used more broadly - e.g., PubPeer considers itself an "online platform for post-publication peer review" - however, I agree that the terminology might be confusing and so have changed the wording to "Open platforms (“decoupled review”)". The point about open platforms being a fringe trait is valid (I've added text to the open platforms section in the discussion to strengthen the acknowledgement of this) - it was included simply because it was observed to be part of the two definitions cited.

*TB: “p11 – Conclusion. I don’t believe the author presents a unified definition of open peer review, for all the reasons discussed above, but he does present most of the traits that together come under the umbrella term.”*

TRH: I have removed the term "unified" as this is no doubt contentious

# Peer review: Referee response for version 1

**DOI:** 10.5256/f1000research.12273.r22299

**Published:** 2017-05-08

**Authors:** Richard Walker

**Affiliations:** 1 Blue Brain Project, Swiss Federal Institute of Technology in Lausanne, Geneva, Switzerland

**General**

 This is a useful, well-written article that helps to clarify some of the “fuzziness” concerning the concept of “Open Peer Review”.

 The author makes a systematic search of the literature, fully and correctly detailing his methods in the Supplementary Materials. His main conclusion is that  “Open peer review is an umbrella term for a number of overlapping ways that peer review models can be adapted in line with the ethos of Open Science…”. The data from the systematic review fully justifies this conclusion. The references are comprehensive and up to date.

 On this basis, I believe the quality of the article is already sufficient to justify publication. I would like, nonetheless to suggest some possibilities for improvement.

**Weak conclusions**

 The author’s conclusions, while correct, are weak. The rapid growth in references to Open Peer Review in the literature suggests that interest in OPR is growing rapidly. It would be useful to point this out. It would also be useful to point out that 110/122 references in his survey talk about “Open Identities” and 72 talk about Open Reports”. This suggests to me that the core sense of Open Peer Review lies precisely in the use of Open identifies and Open Reports and that other aspects are more peripheral. If this were my article (which it is not) I would make this core/periphery distinction more explicit. The author observes correctly that there is still very little evidence about the effectiveness or otherwise of different forms of Open Peer Review. This is another issue that it would be good to bring out in the conclusions.

**“Power distribution” -**

 The author claims that the configurations of OPR traits “follow a power-law distribution”. Readers will understand what he means. However a power law is a functional relationship between two quantities – and here I see only one (the number of configurations). Power laws play no further part in the author’s argument. So I suggest it would be better to avoid the term. What the author could say, correctly, is that there are a couple of very common configurations and a lot of rarer ones. This links to the idea of a “core” and “peripheral” concepts of OPR.

**Reasons for open reports**

 The author correctly argues that Open Identities provide an incentive to reviewers to do their work thoroughly. I suggest that the same applies to “Open Reports”. No reviewer wants to expose himself/herself as lazy or blatantly unfair.

**Detailed points.**

  

- P4: I suggest the author replaces “Unaccountability” with “Lack of accountability”
- P6: In the methods, there seem to be two literature surveys, the first by OpenAire (never mentioned again in the rest of the article), the second by the author. The author should clarify exactly who did what and how he used the OpenAire search
- P6: The text at the top of column 2 starts in the middle of a sentence. I think something is missing.
- P7: In Figure 4, it is not clear what is the metric. Is it the number of Journal Articles/Grant proposals etc. or is it the number of distinct definitions found in journals etc? It would be good to clarify what is meant by “Data,Journal Articles”
- P7: The author writes that “for the literature offers a total of 22 distinct configurations of seven traits, effectively meaning that there are 22 different definitions of OPR in the literature." In reality he found 22 configurations in his, necessarily limited survey. I am certain the literature contains many more. I suggest he corrects his initial statement to make this clear.
- P7: The definition of “Open identities”, “Open Reports” etc. is given in the discussion. I suggest it would be useful to insert the definitions, earlier, immediately after the introduction of the schema (column 2 p 7)
- P8: It might be worth mentioning that some publishers (like Frontiers) favor a system of Open Peer Review which publishes reviewers’ names, only when articles are accepted, thereby avoiding the risk of self-censorship by critical reviewers. 

I have read this submission. I believe that I have an appropriate level of expertise to confirm that it is of an acceptable scientific standard.

# Response

**Published:** 2017-08-31

**Authors:** Tony Ross-Hellauer

**Affiliations:** OpenAIRE / Uni. Goettingen, Germany

***Richard Walker:*** *“This is a useful, well-written article that helps to clarify some of the “fuzziness” concerning the concept of “Open Peer Review”. The author makes a systematic search of the literature, fully and correctly detailing his methods in the Supplementary Materials. His main conclusion is that “Open peer review is an umbrella term for a number of overlapping ways that peer review models can be adapted in line with the ethos of Open Science…”. The data from the systematic review fully justifies this conclusion. The references are comprehensive and up to date. On this basis, I believe the quality of the article is already sufficient to justify publication. I would like, nonetheless to suggest some possibilities for improvement.”*

**Tony Ross-Hellauer:** I'd like to personally thank the reviewer for their time and care in undertaking this review, which has helped strengthen the paper, especially with regards to the suggestions for strengthening the conclusion.

***RW:*** *Weak conclusions - The author’s conclusions, while correct, are weak. The rapid growth in references to Open Peer Review in the literature suggests that interest in OPR is growing rapidly. It would be useful to point this out. It would also be useful to point out that 110/122 references in his survey talk about “Open Identities” and 72 talk about Open Reports”. This suggests to me that the core sense of Open Peer Review lies precisely in the use of Open identifies and Open Reports and that other aspects are more peripheral. If this were my article (which it is not) I would make this core/periphery distinction more explicit. The author observes correctly that there is still very little evidence about the effectiveness or otherwise of different forms of Open Peer Review. This is another issue that it would be good to bring out in the conclusions.*

**TRH: **I agree that the conclusion was weak and welcome the suggestions for improvement. I have written an extended conclusion which I believe addresses all these points.

*RW: ““Power distribution” - The author claims that the configurations of OPR traits “follow a power-law distribution”. Readers will understand what he means. However a power law is a functional relationship between two quantities – and here I see only one (the number of configurations). Power laws play no further part in the author’s argument. So I suggest it would be better to avoid the term. What the author could say, correctly, is that there are a couple of very common configurations and a lot of rarer ones. This links to the idea of a “core” and “peripheral” concepts of OPR.”*

TRH: Text changed to "The distribution of traits shows two very popular configurations and a variety of rarer ones ..."

*RW: “Reasons for open reports - The author correctly argues that Open Identities provide an incentive to reviewers to do their work thoroughly. I suggest that the same applies to “Open Reports”. No reviewer wants to expose himself/herself as lazy or blatantly unfair.”*

TRH: Agreed - I have added text to this effect: "It could also increase review quality, as the thought of their words being made publicly available could motivate reviewers to be more thorough in their review activities. "

*RW: “P4: I suggest the author replaces “Unaccountability” with “Lack of accountability””*

TRH: Text changed as suggested.

*RW: “P6: In the methods, there seem to be two literature surveys, the first by OpenAire (never mentioned again in the rest of the article), the second by the author. The author should clarify exactly who did what and how he used the OpenAire search”*

TRH: There was only one lit review, done by the main author as part of the OpenAIRE project - for clarity, I've changed the reference to OpenAIRE to the first person singular ("I")

*RW: “P6: The text at the top of column 2 starts in the middle of a sentence. I think something is missing.”*

TRH: I've changed the structure of this sentence to be less confusing: "Sixty-eight percent (n=83) of the 122 definitions identified were explicitly stated, 37.7% (n=46) implicitly stated, and 5.7% (n=7) contained both explicit and implicit information."

*RW: “P7: In Figure 4, it is not clear what is the metric. Is it the number of Journal Articles/Grant proposals etc. or is it the number of distinct definitions found in journals etc? It would be good to clarify what is meant by “Data,Journal Articles””*

TRH: This is explained in the text at the bottom of p5 of v1: "Meanwhile, regarding the target of the OPR mentioned in these articles (Figure 4), most were referring to peer review of journal articles (80.7%), with 16% not specifying a target (16%), and a small number of articles also referring to review of data, conference papers and grant proposals."

*RW: “P7: The author writes that “for the literature offers a total of 22 distinct configurations of seven traits, effectively meaning that there are 22 different definitions of OPR in the literature." In reality he found 22 configurations in his, necessarily limited survey. I am certain the literature contains many more. I suggest he corrects his initial statement to make this clear.”*

TRH: Added text to sentence to read "in the literature examined here."

*RW: “P7: The definition of “Open identities”, “Open Reports” etc. is given in the discussion. I suggest it would be useful to insert the definitions, earlier, immediately after the introduction of the schema (column 2 p 7)”*

TRH: Text added as suggested.

*RW: “P8: It might be worth mentioning that some publishers (like Frontiers) favor a system of Open Peer Review which publishes reviewers’ names, only when articles are accepted, thereby avoiding the risk of self-censorship by critical reviewers.”*

TRH: This is a good suggestion.
